# A mass occurrence of pteropods (*Limacina* spp.) drove a pronounced peak in zooplankton biomass in Atlantic water in the Barents Sea in 1994

**DOI:** 10.1093/plankt/fbaf012

**Published:** 2025-03-27

**Authors:** Hein Rune Skjoldal, Espen Bagøien, Monica Bente Martinussen

**Affiliations:** Institute of Marine Research, Ecosystem Processes Research Group, PO Box 1870, Nordnes, N-5817 Bergen, Norway; Institute of Marine Research, Plankton Research Group, PO Box 1870, Nordnes, N-5817 Bergen, Norway; Institute of Marine Research, Plankton Research Group, PO Box 1870, Nordnes, N-5817 Bergen, Norway

**Keywords:** pteropods, *Limacina*, *Calanus finmarchicus*, zooplankton biomass, size fractionation

## Abstract

Zooplankton have been monitored on autumn cruises in the Barents Sea since the late 1980s. The time series shows a pronounced peak in zooplankton biomass in the inflow region of Atlantic water in 1994. The mean biomass was ~ 20 g dry weight m^−2^, which is more than twice the long-term average, and showed an atypical composition with dominance of the small size fraction (<1 mm). Analysis of stored samples revealed that the high biomass event in 1994 was due to a mass occurrence of two species of *Limacina* pteropods (*Limacina retroversa* and *Limacina helicina*) dominated by small juveniles < 1 mm in diameter. High biomass in the Atlantic inflow region also in 1995 was due to a strong but delayed summer generation of the dominant copepod *Calanus finmarchicus*. Estimated biomass of copepods (from numbers and individual weight by species and stage) was strongly dominated by *C. finmarchicus* in both years (~90%). The average biomass of *Limacina* spp. in 1994 was ~ 7 g dw m^−2^, estimated to be mainly in the small fraction, and contributed to the 1994 peak on top of a “typical” biomass of *C. finmarchicus*. The results contribute to a better understanding of the Barents Sea ecosystem.

## INTRODUCTION

**Fig. 1:**
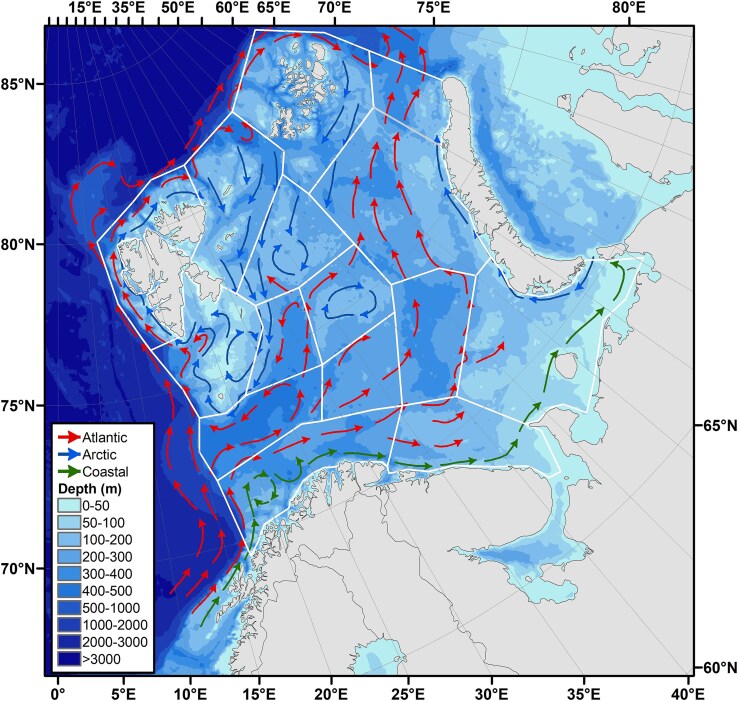
Map of the Barents Sea with bottom topography (color coded), water circulation and division into subareas or polygons. The water circulation is shown schematically with currents of coastal water (the Norwegian Coastal Current), Atlantic water and Arctic water. Atlantic water flows north along the slope and shelf of Norway as the Norwegian Atlantic Current, which splits into two branches, with the North Cape Current flowing east into the Barents Sea, and the West Spitsbergen Current flowing north on the west side of Svalbard (Skagseth *et al.*, 2008). The polygons are broadly based on topography and associated changes in oceanography (see Fig. S1 for names of the polygons and location of the four Atlantic polygons).

The Barents Sea is a high-latitude ecosystem located at the northwestern corner of Europe (Ozhigin *et al.*, 2011). It is a wide (~1.5 million km^2^) and relatively deep shelf-sea (average depth ~ 240 m), bordered by deep ocean basins to the west and north (Fig. 1). The Barents Sea is part of the Atlantic gateway to the central Arctic Ocean (Ingvaldsen *et al.*, 2021, 2024), and as such it constitutes a transition between boreal and Arctic biogeographical zones. The oceanography of the Barents Sea is characterized by inflow and throughflow of relatively warm Atlantic water (~5–6°C), which is cooled and partially transformed into Arctic water *en route* from the entrance in southwest to the exit to the Arctic Ocean in northeast (Fig. 1; Ozhigin *et al.*, 2011; Skagseth *et al.*, 2008, 2020; Ingvaldsen *et al.*, 2021).

The Barents Sea has been on a warming trend since ~ 1980, with an increase in temperature of nearly 2°C (Fig. S2). The temperature increase has affected all areas of the Barents Sea, reflecting the dominant influence of the Atlantic water (Skagseth *et al.*, 2020), and has been associated with a pronounced decrease in the extent of sea ice in winter (Onarheim *et al.*, 2018). Superimposed on the increasing trend, the temperature of the inflowing Atlantic water has fluctuated between warmer and colder years (Fig. S2).

The zooplankton of the Barents Sea is dominated by two species of *Calanus* copepods: *C. finmarchicus*, a boreal species in the Atlantic water of the southern Barents Sea, and *C. glacialis* in the Arctic waters of the northern Barents Sea (Melle and Skjoldal, 1998; Falk-Petersen *et al.*, 2009). These two species are estimated to make up ~ 80% of the total biomass of mesozooplankton (Aarflot *et al.*, 2018; Skjoldal and Aarflot, 2023).

The Institute of Marine Research in Norway (IMR) has monitored zooplankton in the Barents Sea on autumn surveys since the late 1980s, following on from an intensive period of research on zooplankton since 1979 (Skjoldal, 2024). The procedure has been to measure biomass in three size fractions in one half-sample, while the other half is preserved for taxonomic analysis. The time series from the monitoring has revealed a pronounced peak in zooplankton biomass in the inflow region of Atlantic water in the southwestern Barents Sea in 1994 (Dalpadado *et al.*, 2020; Skjoldal *et al.*, 2022). The mean biomass calculated for a large area of the western and central Barents Sea has varied typically around 6–8 g dry mass (or dry weight, dw) m^−2^ in most years, with a minimum of 4 g dw m^−2^ in 1990 and a maximum of 13 g dw m^−2^ in 1994 (Dalpadado *et al.*, 2020). The biomass peak in 1994 was especially pronounced in the inflow region of Atlantic water in the southwestern Barents Sea where the mean biomass for subareas (or polygons, Fig. 1, Fig. S1) was up to or even exceeding 20 g dw m^−2^ (Fig. S3; Skjoldal *et al.*, 2022). The 1994 biomass peak differed from the general pattern of the time series by showing an unusually high contribution of the small size fraction (Skjoldal *et al.*, 2022; Skjoldal and Sperfeld, 2024).

The objective of this study is to establish what drove the exceptional peak in zooplankton biomass in 1994. To address this question, we made a retrospective analysis by using archived half-samples corresponding to the samples used to describe patterns of biomass. The high total biomass in the Bear Island Trench (BIT), the main area for inflow of Atlantic water from the adjacent Norwegian Sea (Fig. 1), persisted in 1995 (Fig. S3), with a similarly strong contribution of the small fraction (~10 g dw m^−2^ to the total of ~ 20 g dw m^−2^; Skjoldal *et al.*, 2022, their Fig. 5a). We therefore included samples from 1995 to examine if the high biomass event this year was due to the same phenomenon as in 1994.

We show that small pteropods of genus *Limacina* contributed to the high biomass of the small size fraction and the high total zooplankton biomass in the Atlantic inflow region in 1994. There are two species of *Limacina* in the Barents Sea—*Limacina helicina*, which is an Arctic-subarctic species, and *Limacina retroversa*, which is a more boreal and temperate species (van der Spoel, 1972; Lalli and Gilmer, 1989). They belong to the group of thecosome (shelled) pteropods and are called sea-butterflies from their fluttering flaps of the wing-like structures of their gastropod foot (pteropod) as they swim. *L. helicina* is the larger of the two with a size (diameter) up to 10–13 mm, whereas *L. retroversa* is smaller, reaching 2–3 mm in size (Lalli and Gilmer, 1989). The shell of *L. retroversa* is more pointed compared to the flatter (more compressed) shell of *L. helicina*, and its shell spirals the “opposite” way (which is why it is called *retroversa*; Fig. S4). Both species have wide geographical distributions and co-occur in the Atlantic water mass of the southern Barents Sea and in waters at Svalbard (Eiane and Tande, 2009; Boissonnot *et al.*, 2021).

There is currently a large interest in the *Limacina* species due to their perceived sensitivity and vulnerability to climate change, including potential effects of ocean acidification on their calcareous shells (Comeau *et al.*, 2012; Lischka and Riebesell, 2012; Bednaršek *et al.*, 2016). Despite this interest, there is still considerable uncertainty about the life histories and life cycles of *Limacina* species (Wang *et al.*, 2017; Gardner *et al.*, 2023). *L. helicina* and *L. retroversa* have complex taxonomy and are considered to form species complexes (Lalli and Gilmer, 1989; Jennings *et al.*, 2010). *L. helicina* has been described with two subspecies, *L. helicina helicina* in northern waters and *L. helicina antarctica* in the Southern Ocean, each with several morphotypes or forms (*acuta*, *helicina* and *pacifica* for the northern subspecies *L. helicina helicina*). The two subspecies have been shown to be genetically distinct (Hunt *et al.*, 2010), and the Antarctic subspecies is considered a separate species *Limacina rangii* (Gardner *et al.*, 2023). *L. retroversa* occurs with two recognized forms in the northern hemisphere (*balea* and *retroversa*).

The *Limacina* species feed as broad-spectrum microplankton grazers (Mackas and Galbraith, 2012), using an external mucus feeding web to collect phytoplankton, microzooplankton and detritus (Gilmer and Harbison, 1986; Harbison and Gilmer, 1992). These pteropods are hermaphroditic and produce floating egg masses that hatch into small veliger larvae (Paranjape, 1968). The veligers grow into small juveniles that develop their external shell when they are ~ 0.5 mm in size (Lalli and Wells, 1978). At this stage, *L. helicina* and *L. retroversa* are difficult to distinguish morphologically before the shells are sufficiently developed to tell them apart (Lischka *et al.*, 2011). The *Limacina* species reproduce once or twice a year, with peak spawning in spring and/or autumn. *L. helicina* was found to reproduce in late summer in waters around Svalbard in the northern Barents Sea (Gannefors *et al.*, 2005; Boissonnot *et al.*, 2021). The feeding mode apparently allows them to overwinter as small juveniles, presumably nourished by detritus and heterotrophic microplankton, in addition to using stored lipid reserves.

We have used the new species counts to calculate biomass of copepods (Skjoldal and Aarflot, 2023) and pteropods, and their contributions to the measured biomass values in 1994 and 1995. For pteropods (*Limacina* species), we measured their size and used relationships between weight and width to estimate biomass. We also use the data on size frequency distribution of the small juvenile *Limacina* to provide information on their life histories. In addition, we present information from the IMR database on the abundance of pelagic gastropods (mainly *Limacina*) in the Barents Sea for the years 1995–2022 to illustrate the exceptionally high abundance of *Limacina* in the biomass peak in 1994, and to shed further light on their life history.

## METHOD

### Sampling

**Fig. 2:**
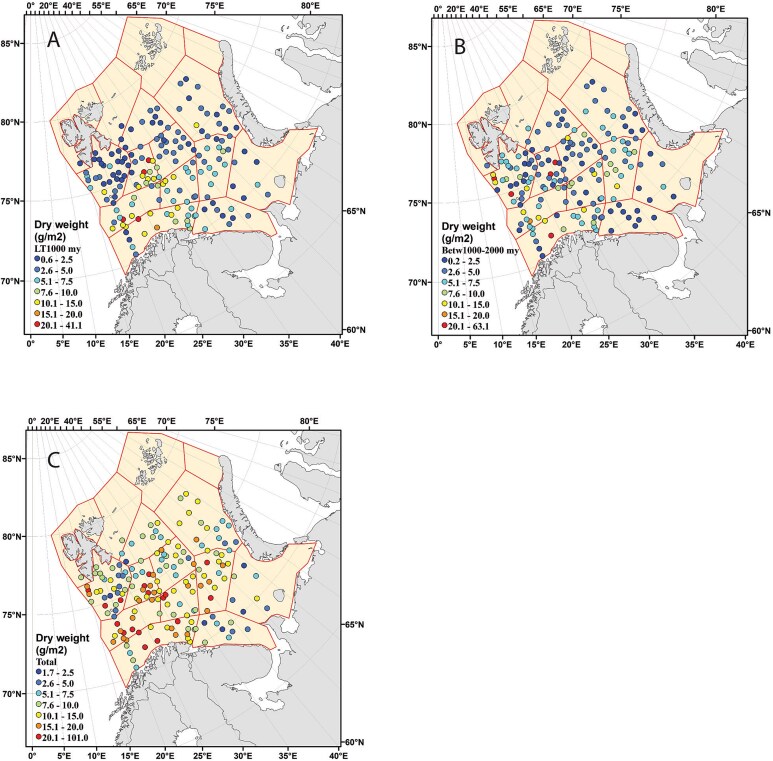
Distribution of zooplankton biomass (g dw m^−2^) in the Barents Sea in autumn 1994 for (**A**) the small (< 1 mm) and (**B**) medium (1–2 mm) size fractions, and (**C**) total biomass.

Zooplankton samples were collected on autumn cruises (mid-August to early October) with RVs “G.O. Sars” and “Johan Hjort” in 1994 and 1995. Sampling covered much of the Barents Sea (including the Russian sector) north to ~78^o^N in both years (see Fig. 2), with a total number of 175 (1994) and 117 (1995) sampling stations. Samples were collected with vertical hauls with WP-2 net (0.25 m^2^, 180 μm mesh), from near bottom (~10 m) to the surface. Upon retrieval and hosing of the net with seawater, the content of a cod-end was divided into two halves with a Motoda plankton splitter, for determination of dry weight biomass and species composition, respectively (Skjoldal *et al.*, 2013; Hassel *et al.*, 2020).

### Biomass

The half-sample for biomass determination was fractioned by successive wet sieving through 2000, 1 000, and 180 μm meshed screens. The contents on each screen were transferred to pre-weighed aluminum trays, dried at 65°C for 24 h or more, and weighed in the laboratory on shore after the cruise. The results are expressed as g dry mass (or dry weight, dw) per m^2^ by multiplying sample weight by 8 (2×4 to account for initial splitting of sample and mouth area of the net, respectively). The WP-2 net was operated without a flow meter, and the calculation assumes 100% filtration efficiency. Clogging of the net rarely occurs in autumn when chlorophyll levels are generally low (Skjoldal and Aarflot, 2023).

### Taxonomic analysis

The half-samples for species counts were preserved with buffered (borax) formaldehyde and stored in a sample repository at IMR. We selected 23 samples from 1994 that included sampling stations with recorded high biomass of the small size fraction as well as high total zooplankton biomass. The samples represented four polygons in the southwestern Barents Sea: South-West (SW), BIT, Thor Iversen Bank (TIB) and Hopen Deep (HD; Fig. 1, Fig. S1, Table S1). The inflow of Atlantic water (including the Norwegian Coastal Current) takes place through the SW and BIT polygons and continues with a main branch through the TIB polygon and a smaller branch into the HD polygon (Fig. 1). We call these four polygons collectively as Atlantic polygons (Fig. S1). Eleven samples with high recorded zooplankton biomass were selected in 1995, representing stations from three of the Atlantic polygons (SW, BIT and TIB) as well as the North-East polygon (Table S1).

Samples were analyzed with the standard routine procedure for species counts at IMR (Hassel *et al.*, 2020). The method is adaptive using subsampling and aims at counting at least 100 individual copepodites of *C. finmarchicus* and/or *C. glacialis* which are usually the dominant species in terms of biomass (Skjoldal and Aarflot, 2023). Large zooplankton individuals retained on a 2-mm screen are counted in the whole half-sample, whereas smaller forms are counted in subsamples. Copepodite stages of *C. finmarchicus* were often counted in subsample fractions of 1/128 or 1/256, but also in lower (1/32, 1/64) or higher (1/512, 1/1024) subsample fractions when abundance was either low or very high. Small pteropods (which were abundant in 1994) were typically counted in 1/256 subsample fraction.

*Calanus* species (*C. finmarchicus*, *C. glacialis*, and *C. hyperboreus*) were counted for each of the six copepodite stages separately. *Metridia*, *Pseudocalanus* and *Paraeuchaeta* species were counted at the genus level for two groups of copepodites (CI-III and CIV-V) and at the species level for *Metridia* (*M. longa* and *M. lucens*) and *Paraeuchaeta* (*P. norvegica*) for the adult stage (females and males). Other copepods (e.g. *Oithona*, *Microcalanus*, *Acartia*) were counted at the genus level for copepodites combined (for small species it is mostly the older copepodites that are retained by the 180 μm mesh net; Skjoldal *et al.*, 2013). Other zooplankton taxa were counted at species level (e.g. *Aglantha digitale*, *Clione limacina*), genus level (e.g. *Eukrohnia*, *Sagitta*) or higher taxonomic level (e.g. echinoderm larvae, polychaete larvae).

Attention was given to pteropods (*Limacina* spp.) since logbooks from the cruises in 1994 noted high abundances when samples were collected and processed onboard. The two species of *Limacina* in the Barents Sea, *L. retroversa* and *L. helicina*, are difficult to tell apart when small (<0.6 mm diameter) and were then counted as undetermined *Limacina* spp. From a size of ~ 0.6 mm diameter, the shell was sufficiently developed to allow the species to be recognized and counted separately (see photo in Fig. S4 in Supplementary material). The size of the individuals was measured with an ocular micrometer as the diameter of the shell (Kobayashi, 1974). A subsample of typically more than 100 individuals (mean 185, range 85–298) was sized and used to determine the proportions of small undetermined *Limacina* spp. juveniles, and larger juveniles identified as the two *Limacina* species. The smallest mark on the ocular scale (at 16x magnification) was 0.06 mm, and individuals were sized to the nearest mark.

### Estimation of biomass of copepods and Limacina pteropods

The biomass of copepods was calculated from abundance numbers from the species counts multiplied by average weight by species and copepodite stages following Skjoldal and Aarflot (2023; see their Table S1 in Supplementary data). An exception was that the weights for the stages of *C. finmarchicus* were reduced by 30% as suggested by Skjoldal and Aarflot (2023). The estimated biomass of copepods was partitioned among the three size fractions dependent on the average size of taxa, based on the results of Skjoldal (2021) and following Skjoldal and Aarflot (2023). Proportions of biomass allocated to the three fractions for species and stages of copepods were as given by Skjoldal and Aarflot (2023) in their Table S1.

The biomass of *Limacina* pteropods was determined from size frequency distributions for each of the three categories representing undetermined small juveniles, and larger individuals identified as *L. helicina* and *L. retroversa*, respectively. We compared published relationships for individual dry weight versus diameter for *L. helicina* (Gannefors *et al.*, 2005), *L. retroversa* and *L. helicina* (Conover and Lalli, 1972, with weight recorded as ash-free dry weight), and *L. helicina antarctica* (Bednaršek *et al.*, 2012). There were considerable differences between these relationships (Fig. S5), which we believe are at least partly due to the regressions being determined for different size ranges of *Limacina* species. The discrepancies were particularly large in the low size range < 1 mm diameter, which is where the bulk of our samples lie. As an alternative, we calculated hypothetical volume of *Limacina* from the equation of a sphere with the recorded diameter. Assuming a density of 1.0 (for weight per volume, 1 cm^3^ = 1 g wet weight) and a dry weight content of 20% (80% water content) for juvenile *Limacina* (Kiørboe, 2013; Ikeda, 2014), volume was converted to dry weight by factor 0.2. This volume-based estimate of individual weight was lower by factor 0.15–0.40 for the size range 0.3–1.0 mm in diameter compared to Gannefors *et al.* (2005), and higher by factor 3.3–2.6 for the same size range compared to Conover and Lalli (1972) for *L. retroversa* (Fig. S5). We used volume-based estimate of dry weight for the undetermined small juveniles and *L. retroversa*, while we reduced the biomass to 2/3 of volume for the more compressed *L. helicina*.

The estimated biomass of *Limacina* pteropods was partitioned among the small and medium fractions using a cut-off limit of 1 mm in diameter, equal to the screen mesh size used to separate the biomass samples. For copepods with elongated body shape, retention by the 1-mm screen depends on a combination of their width and length, with separation taking place at width between ~ 0.4 and 0.8 mm (Skjoldal, 2021). For the globular-shaped *Limacina* pteropods, we assumed that the separation would be determined by the diameter, similar to the situation with a towed zooplankton net where median retention is at width equal to the mesh size (Skjoldal *et al.*, 2013).

### Data analyses

Descriptive summary statistics and some plots of results were generated with Microsoft Excel. Box-whisker diagrams and scatter plots with regression lines were produced with R v. 4.3.1 (R Core Team, 2023) using the package ggplot2 (Wickham, 2016). The statistical significance of differences between two means was tested with R applying two-sided *t*-tests (not assuming equal variances) on log10-transformed biomass data (with the low value 0.01 (g dw m^−2^) added to account for occasional zero observations of biomass for the large size fraction). Two-way ANOVA with an interaction-term was used to test differences of biomass (log10-transformed after adding the value of 0.01) of the 3 size fractions and total between the two years (1994 and 1995) and two sets of polygons (Atlantic and others, Fig. S1). The ANOVA was computed for unbalanced design (Type-III test) applying the Anova-function of the R-package “car” (Fox and Weisberg, 2019).

## RESULTS

### Biomass

High biomass values in 1994 for the small and medium size fractions were largely confined to the four Atlantic polygons in the southwestern Barents Sea (SW, BIT, HD and TIB; Fig. 2A and B), as was also the case for total biomass (sum of the 3 fractions, Fig. 2C). Biomass in 1995 showed similar patterns with some high biomass values in the Atlantic polygons, but with high values also in the Southeastern Basin and North-East polygons located further east (Fig. S6).

The biomass values for the 3 size fractions and their sum as total biomass showed highly right-skewed distributions on linear scale with scattered high values as statistical outliers in the high end (Fig. S7A). Log-transformation of the data gave balanced (log-normal-like) distributions (Fig. S7B). The maximum recorded total biomass in 1994 was 101 g dw m^−2^ (stn 992 in HD), which is the record over > 30 years of sampling in the Barents Sea (>4 000 WP-2 samples). The highest value in 1995 was 33 g dw m^−2^ (stn 842 in BIT).

**Fig. 3:**
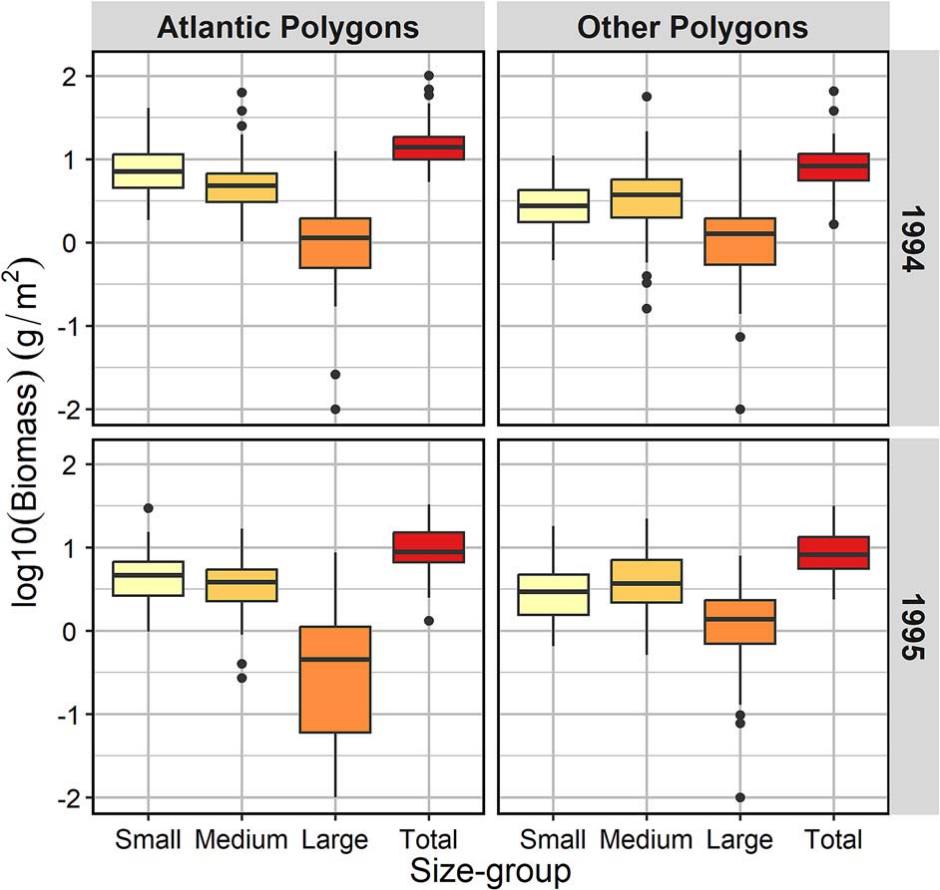
Box-whisker plots of biomass (log10 g dw m^−2^ + 0.01) in Atlantic (SW, BIT, TIB, HD) and “other” polygons in 1994 (upper panels) and 1995 (lower panels; see maps in Fig. 2 and Fig. S6) for three size fractions and total biomass. The figures show median values (horizontal line), 25 and 75% quantiles (boxes), and range of data (vertical lines) apart from observations defined as statistical outliers (dots).

The biomass values showed statistically significant differences between the two years and between the two sets of polygons, as well as for the interaction between years and polygons (two-ways ANOVA, Table S2). The effects were strongest for the small size fraction and total biomass and least for the medium fraction. The highest recorded biomass for the small size fraction was 41 g dw m^−2^ in 1994 (stn 1 160 in SW). The mean biomass for the small fraction was 9.1 g dw m^−2^ for the Atlantic polygons in 1994, significantly higher than the biomass of the small fraction in the “other polygons” (Fig. 3, Table I). The mean biomass of the medium fraction in 1994 was also higher for Atlantic polygons (7.0 vs 4.8 g dw m^−2^) as was the total biomass (17.8 vs 9.7 g dw m^−2^), the latter driven largely by the higher biomass of the small fraction (Table I). The biomass values for the Atlantic polygons in 1995 were less extreme, although the biomass of the small fraction was still significantly higher than for the “other polygons” (Table I). The biomass values for the “other polygons” were similar between 1994 and 1995 (Fig. 3, Table I).

**Table I:** *Statistical summaries of zooplankton biomass (g dw m^−2^) in 3 size fractions and total for Atlantic and “other” polygons in 1994 and 1995 with mean values and standard deviations (SD), and two-tailed* t*-test for difference between values in Atlantic and “other” polygons. The tests were performed on log10 (biomass+0.01)*

Fraction/year	Atlantic polygons			Other polygons			*t-*test		
	*Mean*	*SD*	*# obs*	*Mean*	*SD*	*# obs*	*t*	*d.f.*	*p*
1994									
Small	9.10	7.01	66	3.24	2.15	109	9.78	130.8	2 × 10^−16^
Medium	7.01	9.14	66	4.84	5.93	109	2.85	148.2	0.005
Large	1.67	2.02	66	1.62	1.76	109	0.00	143.8	1.00
Total	17.78	15.71	66	9.7	7.57	109	6.22	142.9	5 × 10^−9^
1995									
Small	5.62	4.95	51	3.5	2.66	66	3.09	101.6	0.003
Medium	4.43	3.38	51	4.74	3.62	66	−0.66	101.1	0.51
Large	0.83	1.36	51	1.73	1.61	66	−3.54	99.2	0.001
Total	10.88	6.64	51	9.96	5.76	66	−0.44	98.9	0.66

**Fig. 4:**
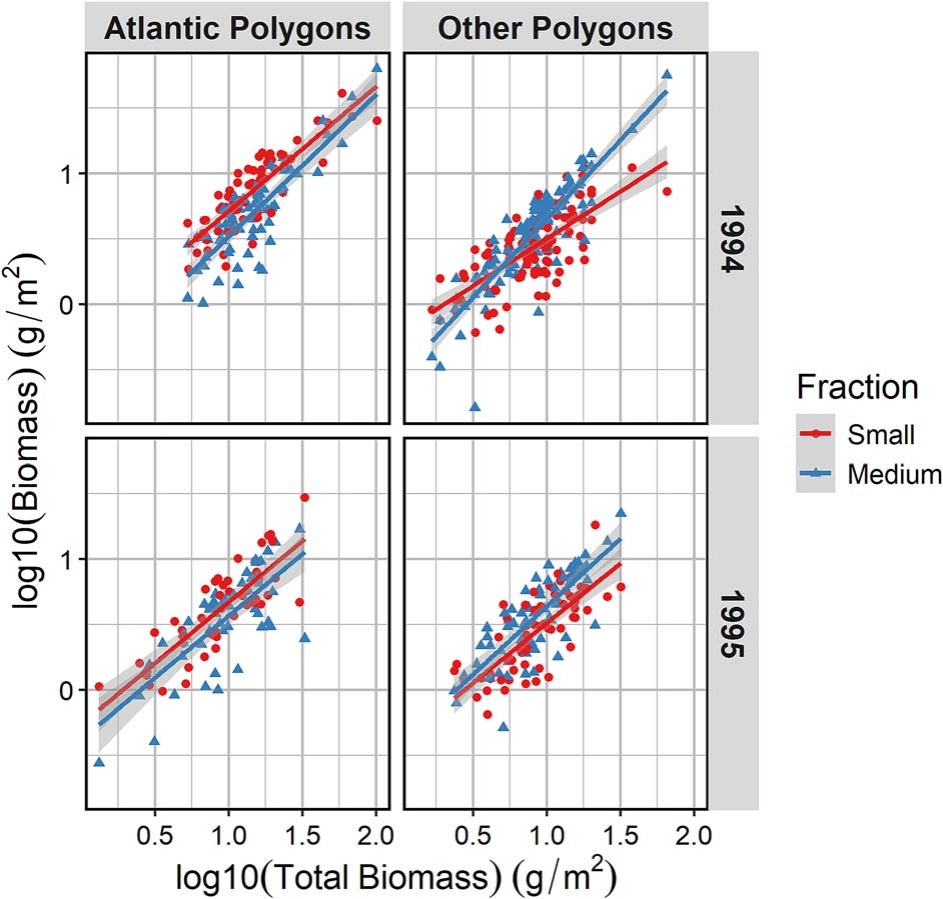
Scatter plots with linear regression lines for biomass of the small and medium size fractions versus total zooplankton biomass for Atlantic and “other” polygons in 1994 (upper panels) and 1995 (lower panels). Values are log10 transformed, and shaded bands are 95% confidence bands for the regression lines (ordinary linear regressions; regression equations are given in Table S3).

The small size fraction contributed ca. half the total biomass in the Atlantic polygons, with similar proportions of 51:39:9% and 52:41:8% for the small, medium and large fractions in 1994 and 1995, respectively. The contribution by the small fraction was lower for the “other” polygons with proportions of 33:50:17% and 35:48:17% for the two years, respectively (based on the average values in Table I). Scatter plots with regression lines showed strict positive relationships for the small and medium size fractions versus total biomass, with similar slopes of the two fractions for Atlantic polygons, demonstrating a persistent trend of higher biomass of the small fraction across the range of values (Fig. 4). For “other” polygons, the slope for the small fraction in 1994 was less steep than the slope for the medium fraction, indicating a shift from dominance of the small fraction at low biomass values to progressively stronger dominance of the medium fraction at high biomass values (Fig. 4).

### Abundance and species composition

**Fig. 5:**
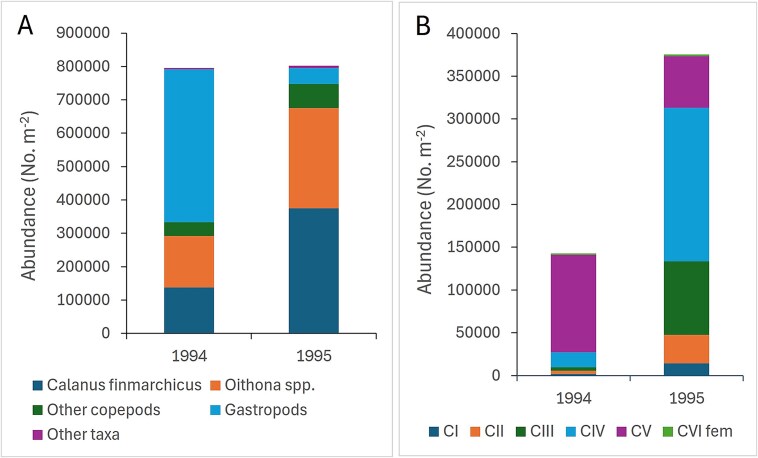
Mean abundance of (**A**) total zooplankton and (**B**) copepodites of *Calanus finmarchicus* for the samples in 1994 and 1995. Other copepods in (A) include *Metridia*, *Pseudocalanus* and *Microcalanus*. See Table II for more information on species composition and variation among stations. The data for the separate stations are shown in Tables S4 and S5. Note that all stations in 1994 and most stations in 1995 were from the Atlantic polygons (Table S1).

The total abundance of zooplankton individuals varied by an order of magnitude across all stations (0.2–2.9 10^6^ individuals m^−2^), with a similar mean abundance of ~ 0.8 10^6^ individuals m^−2^ in both 1994 and 1995 (Table II, Fig. 5A). However, the species composition was markedly different between the two years. In 1994, small pteropods (*Limacina* spp.) made up more than half of the total numbers (458 000 ind. m^−2^), followed by *Oithona* spp. (154 000 ind. m^−2^) and *C. finmarchicus* (138 000 ind. m^−2^). In 1995, young copepodites of *C. finmarchicus* dominated (abundance of all copepodites was 375 000 ind. m^−2^), along with high numbers of *Oithona* spp. (300 000 ind. m^−2^; Fig. 5, Fig. S8). *Limacina* spp. occurred with an order of magnitude lower abundance in 1995 (47 000 ind. m^−2^) compared to 1994.

**Table II:** Statistical summary of abundance of zooplankton taxa (number of individuals per m^2^) for the stations in 1994 (23 stations, Table S1) and 1995 (11 stations). Columns show mean values, SD and minimum and maximum values for the sample series from each year. The data for the separate stations are shown in Tables S3 and S4

Species	Stage	1994				1995			
		Mean	SD	Min	Max	Mean	SD	Min	Max
*C. finmarchicus*	CI	673	1 405	0	6 144	14 522	18 539	0	49 152
*C. finmarchicus*	CII	1 525	4 348	0	20 480	33 094	46 190	0	155 648
*C. finmarchicus*	CIII	3 233	6 243	0	22 528	86 109	131 735	2048	458 752
*C. finmarchicus*	CIV	17 408	10 899	1 152	47 104	179 293	239 634	512	786 432
*C. finmarchicus*	CV	113 831	145 274	7 424	569 344	60 608	47 949	64	135 168
*C. finmarchicus*	CVI	1 686	3 304	0	16 384	1795	1 579	32	4 096
*C. finmarchicus*	All	138 357	153 135	13 440	607 232	375 421	436 374	29 792	1 516 544
*Calanus glacialis*	CII	0	0	0	0	93	309	0	1 024
*C. glacialis*	CIII	22	107	0	512	2 851	7 373	0	24 576
*C. glacialis*	CIV	67	320	0	1 536	70	166	0	512
*C. glacialis*	CV	32	106	0	512	369	924	0	3 072
*C. glacialis*	CVI	3	7	0	24	10	21	0	64
*C. glacialis*	All	123	455	0	2 152	3 393	7 513	0	24 608
*Calanus hyperboreus*	CII	22	107	0	512	17	58	0	192
*C. hyperboreus*	CIII	134	641	0	3 072	193	616	0	2048
*C. hyperboreus*	CIV	77	141	0	512	56	73	0	240
*C. hyperboreus*	CV	63	55	0	192	60	70	0	200
*C. hyperboreus*	CVI	8	17	0	72	10	19	0	64
*C. hyperboreus*	All	304	691	0	3 336	336	650	0	2 232
*Metridia* spp.	CI-III	6 500	15 091	0	69 632	4 026	9 469	0	30 720
*Metridia* spp.	CIV-V	5 849	3 787	256	11 776	2 935	6 126	0	20 480
*Metridia* spp.	CVI	935	1 014	0	4 096	2 679	3 950	0	12 288
*Metridia* spp.	All	13 284	17 453	512	81 920	9 639	14 357	0	51 200
*Paraeuchaeta* spp.	CI-III	17	59	0	256	2	7	0	24
*Paraeuchaeta* spp.	CIV-V	93	52	0	176	91	88	0	224
*Paraeuchaeta norvegica*	CVI	18	19	0	48	6	7	0	16
*Paraeuchaeta* spp.	All	127	74	0	344	99	87	8	224
*Pseudocalanus* spp.	CI-III	0	0	0	0	768	2 463	0	8 192
*Pseudocalanus* spp.	CIV-V	4 108	9 677	0	45 056	26 624	47 558	0	151 552
*Pseudocalanus* spp.	CVI	351	1 491	0	7 168	796	1 059	0	2 560
*Pseudocalanus* spp.	All	4 280	10 902	0	52 224	25 623	46 211	0	152 576
*Microcalanus* spp.		20 013	17 598	1792	61 440	18 897	9 632	0	32 768
*Oithona* spp.		153 578	116 861	7 680	419 840	300 404	134 247	71 680	542 720
*Oncaea* spp.		1 397	4 211	0	20 480	356	542	0	1 360
Other copepods		236	474	0	1 536	926	2 920	0	9 728
Copepod nauplii		1875	3 954	0	16 384	13 009	26 516	0	90 112
Gastropoda		344 420	558 715	19 456	2 732 032	46 848	44 927	256	114 688
*L. helicina*		57 461	81 698	0	317 440				
*L. retroversa*		56 442	95 595	0	331 776				
*Clione limacina*		47	213	0	1 024	10	16	0	40
*Eukrohnia*		146	118	16	448	252	232	56	776
*Sagitta*		138	153	0	544	164	217	16	608
Chaetognatha		234	377	0	1 024	356	650	0	2048
*Aglantha digitale*		36	25	0	80	25	23	0	56
Appendicularians		1725	3 766	0	16 384	3 747	5 566	0	18 688
Meroplankton (larvae)		796	1 241	0	5 120	1 373	2 391	0	6 656
Other		208	480	0	2048	1 087	3 574	0	11 864
Total		795 227	541 626	164 464	2 901 256	802 084	517 414	307 232	2 127 072

The copepodite stage composition of *C. finmarchicus* was markedly different between the two years (Fig. 5B). Stage CV (which is the main overwintering stage) dominated in 1994 (82%) along with some stage CIV (13%). Stage CV dominated across all stations in the 4 Atlantic polygons (Fig. S9), with numbers varying by more than an order of magnitude (from 13 400 to 607 000 for total copepodites m^−2^; Table II). In contrast, the generally higher abundance in 1995 was due to high numbers of stage CIV (48%) along with high numbers also of stage CIII (23%) and even some CII (9%) and CI (Fig. 5B). The total number of copepodites of *C. finmarchicus* varied by nearly 2 orders of magnitude in 1995 (from 30 000 to 1.5 million ind. m^−2^, Table II). High numbers (200 000 copepodites m^−2^, or more) were seen in the three Atlantic polygons (SW, BIT, TIB), while values tended to be lower in the NE polygon, although with similar dominance by younger copepodite stages (Fig. S10).

### Size distribution and abundance of Limacina species in 1994

**Fig. 6:**
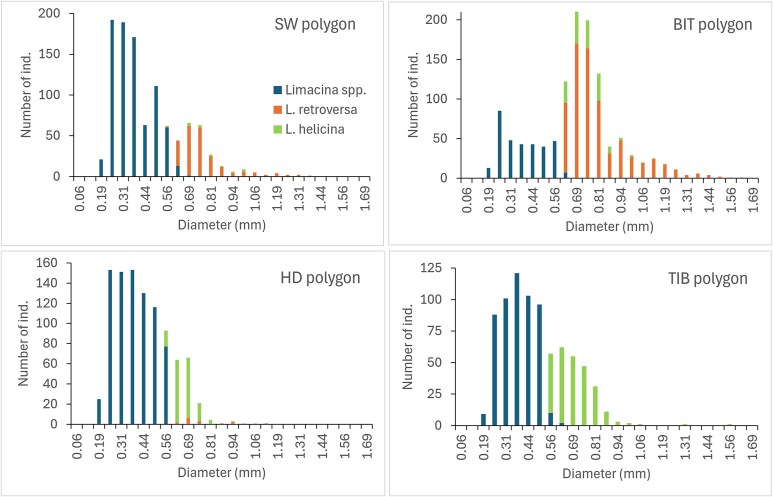
Frequency distributions of size as diameter (mm, x-axis) for the combined numbers of individuals measured at stations in four geographical polygons (SW, BIT, HD, TIB; Fig. S1). Numbers are for unidentified small individuals of *Limacina* spp. (<~0.6 mm) and identified *L. helicina* and *L. retroversa*, respectively.

**Fig. 7:**
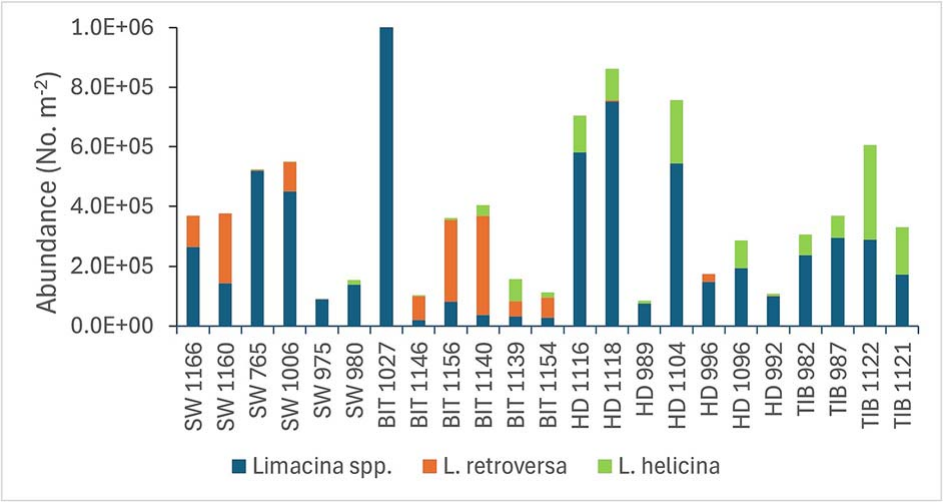
Abundance (no. of individuals m^−2^) of *Limacina* pteropods at 23 sampling stations located in four Atlantic polygons (SW, BIT, HD, TIB) in 1994. The y-axis is cut at 1 million individuals m^−2^ and does not show the maximum value of 2.7 million individuals m^−2^ of mostly small *Limacina* at station 1 027 in BIT.

The pteropods in 1994 were mostly small individuals ranging in size between 0.2 and 1 mm in diameter (Fig. 6). Few individuals were larger than 1 mm, and the largest individual recorded was 2.0 mm (*L. helicina* at station 1 104 in HD polygon). The individuals identified to species (at size > ~ 0.6 mm) were mainly *L. retroversa* in the two southwestern polygons (SW and BIT), while *L. helicina* was the dominant (or only) species at stations further into the Barents Sea (HD and TIB polygons; Figs 6 and 7). The frequency distribution of size for the latter two polygons showed a continuous decline in numbers with increasing size, giving the impression of a cohort of small unidentified juveniles developing into the still small, identified juveniles of *L. helicina*. The situation differed in the other polygons, where the numbers of *L. retroversa* were higher than for the smaller and unidentified *Limacina* spp. in the BIT polygon (Fig. 6). The average size of unidentified *Limacina* spp. varied between 0.3 and 0.5 mm across the 23 stations in 1994, whereas the average sizes of *L. helicina* and *L. retroversa* varied between 0.6 and 0.9 mm (Fig. S11).

The abundance of small unidentified *Limacina* spp. varied by two orders of magnitude, from a low of about 20 000 ind. m^−2^ to a maximum of 2.7 million ind. m^−2^ (station 1 027 in BIT; Fig. 7). This high value was exceptional (the second highest value was 750.000 ind. m^−2^ at station 1 118 in HD). The other values from the BIT polygon tended to be low (mean value 39 000 ind. m^−2^) by an order of magnitude compared to the other 3 polygons (mean values between 248 000 (TIB) and 342 000 (HD) ind. m^−2^; Fig. 7). The abundances of *L. helicina* and *L. retroversa* were comparable in their respective polygons, with averages of ~ 100 000–150 000 individuals m^−2^ for *L. retroversa* in the SW and BIT polygons, and for *L. helicina* in the HD and TIB polygons (Fig. 7).

### Observations of planktonic gastropods 1995–2022

**Fig. 8:**
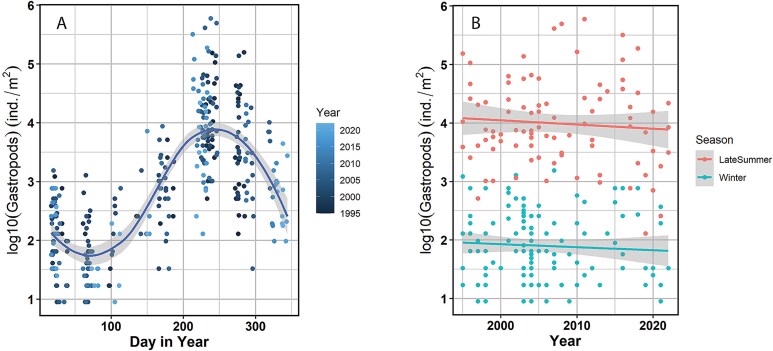
Abundance of planktonic gastropods (mainly *Limacina* spp.) recorded in zooplankton monitoring at the Fugløya-Bear Island transect from 1995 to 2022. (**A**) Seasonal pattern, (**B**) time series 1995–2022. Only samples with positive records of gastropods (56% of all samples) are shown. Abundance (number of individuals m^−2^) is shown on log10-scale. Data in panel (A) are for all the years as identified by color code. Data in panel (B) are for the winter period (January–April) and late summer (August). Curved line in (A) is a *loess* smoother with 95% confidence band. Lines in (B) are linear regression lines with 95% confidence band.

Time series observations with seasonal resolution from the Fugløya-Bear Island transect across the SW and BIT polygons at the southwestern entrance to the Barents Sea have planktonic gastropods recorded in 56% of a total number of 586 samples from the 1995–2022 period. The gastropods belong mainly to genus *Limacina* but have not been identified to species. The abundance of gastropods showed a pronounced seasonal pattern, with low values in winter (<1 000 ind. m^−2^) to early summer (<10 000 ind. m^−2^), and a marked increase in late summer (August) to values typically between 1 000 and 100 000 ind. m^−2^; Fig. 8A). The 28 years of observations reveal no clear trend with time for either the winter (January–April) or late summer (August) periods (Fig. 8B). The mean abundance of gastropods over all samples with positive records (based on log-transformed data) was ~ 800 ind. m^−2^ (log10 value 2.9 with SD = 1.1 log10 unit), with a close to log-normal distribution indicated by low values of skewness (0.25) and kurtosis (−0.66). The maximum recorded abundance was ~ 600 000 ind. m^−2^, and only this and the next highest observation from 1995–2022 exceeded the mean abundance of pteropods in 1994 (458 000 ind. m^−2^, Table II).

### Biomass of copepods and Limacina calculated from abundance

The calculated biomass of copepods was strongly dominated by *C. finmarchicus* in both 1994 and 1995, making up 94 and 91%, respectively (Fig. S12). The rest was made up of *Metridia* spp. (~3%), *Oithona* spp. (~2%), the two other *Calanus* species (*C. glacialis* and *C. hyperboreus* (~0.5–2%), and low amounts (<1%) of other species (including *Paraeuchaeta*, *Pseudocalanus* and *Microcalanus*).

**Fig. 9:**
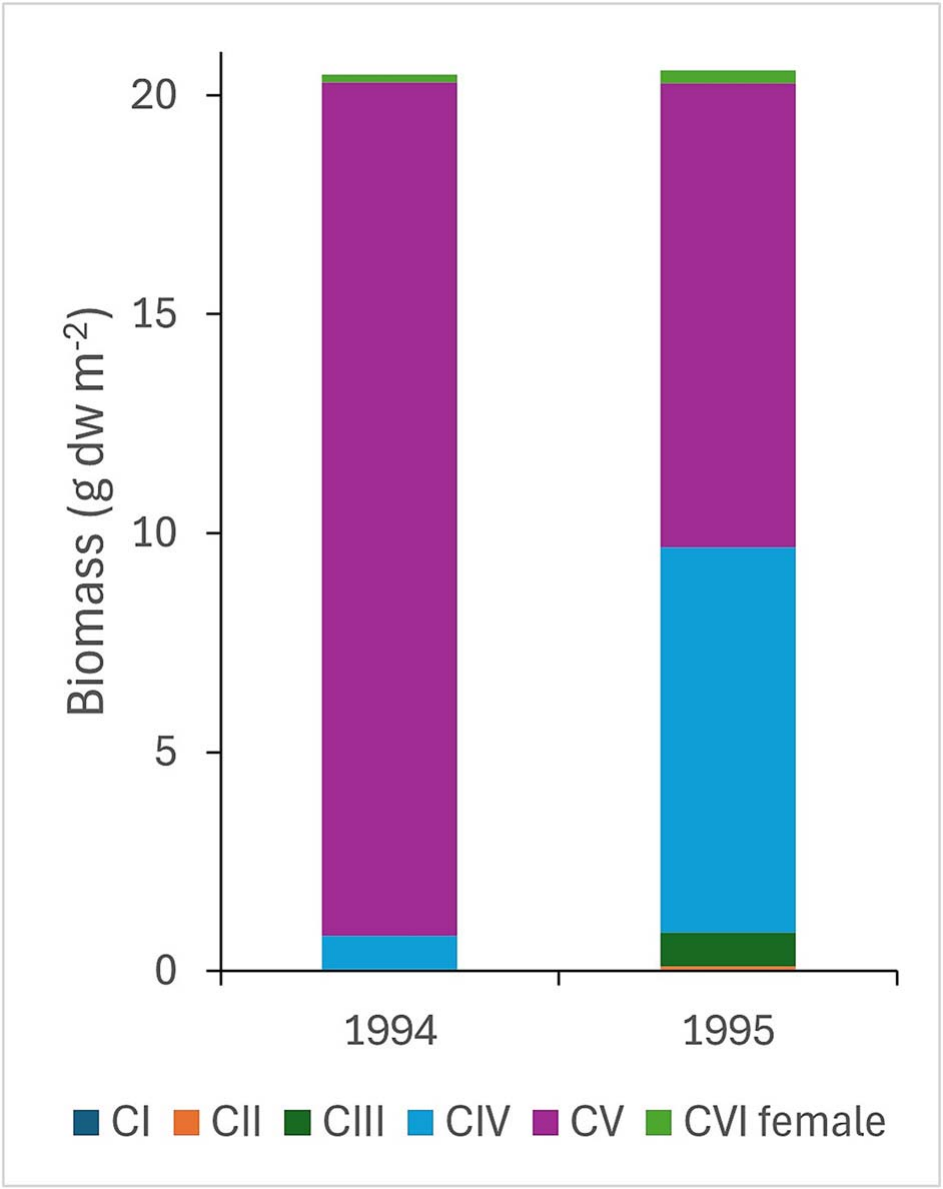
Biomass (g dry weight m^−2^) of *C. finmarchicus* calculated from numerical abundance in 1994 and 1995. Values are averages across all stations (23 in 1994 and 11 in 1995) and are shown for copepodite stages C1 to C6 (adult females). See Figs S13 and S14 for variation across individual stations.

The estimated biomass of *C. finmarchicus* varied by ~ 2 orders of magnitude, with maxima of 102 and 55 g dw m^−2^ in 1994 and 1995, respectively, and mean values of 21 g dw m^−2^ across the analyzed stations for both years (Fig. 9, Figs S13 and S14). In 1994, biomass was dominated by stage C5, which contributed 95% of the calculated biomass of the species (Fig. 9, Fig. S13). The situation in 1995 was different, when stage C4 made up 43% of the biomass on average (Fig. 9, Fig. S14). The three-fold higher numbers of *C. finmarchicus* in 1995 (Fig. 5B) were much reduced in terms of biomass due to the lower weight of young copepodites (Fig. 9).

**Fig. 10:**
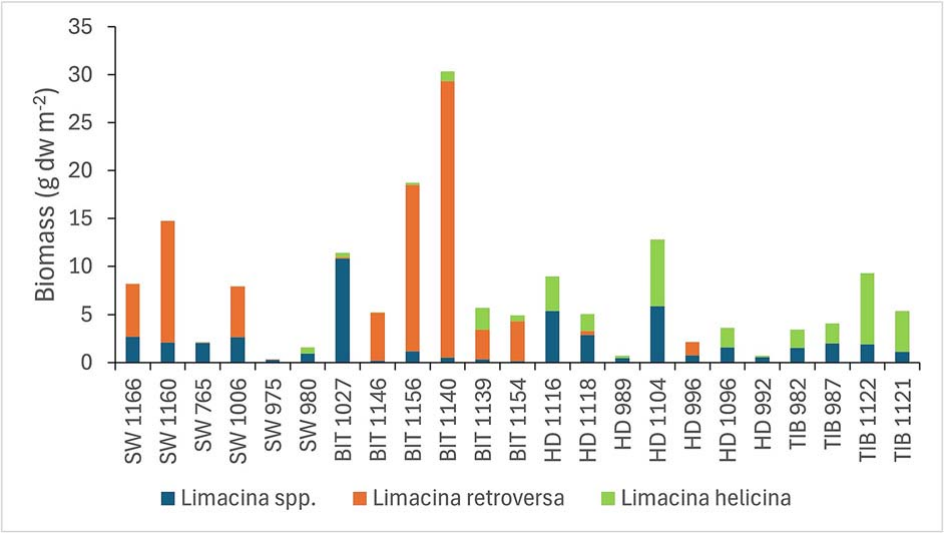
Estimated biomass calculated from numbers of small, unidentified juveniles of *Limacina* spp. and larger (> ~ 0.6 mm) identified juveniles of *L. helicina* and *L. retroversa* at the sampling stations in the four Atlantic polygons (SW, BIT, HD, TIB) in 1994.

Calculated biomass of *Limacina* pteropods (based on volume of a sphere) in 1994 varied by two orders of magnitude, from a low of 0.4 g dw m^−2^ (station 975 in SW) to a maximum of 30 g dw m^−2^ (station 1 140 in BIT; Fig. 10), with an overall mean of 7.3 g dw m^−2^ for the 23 stations in the Atlantic polygons. The average biomass of *L. retroversa* in the SW and BIT polygons was 6.8 g dw m^−2^, while the average biomass of *L. helicina* in the HD and TIB polygons was 2.8 g dw m^−2^. The average biomass of small unidentified juveniles (*Limacina* spp.) was similar in the two sets of polygons (2.0 and 2.2 g dw m^−2^).

The sum of estimated biomass (calculated from numbers) for copepods and *Limacina* pteropods in the Atlantic polygons in 1994 was of the same magnitude as the directly measured biomass in the complementary half-samples for the same stations (mean values of 29 and 28 g dw m^−2^, respectively; Table S6). The two metrics (calculated and measured biomass) were positively and significantly correlated across the 23 stations (Fig. S15, r = 0.87, *P* < 0.001). The biomass of copepods (mainly *C. finmarchicus*) was about three times the estimated biomass of *Limacina* pteropods as average values across the 23 stations in Atlantic polygons in 1994 (22 versus 7 g dw m^−2^). However, in the two southwestern-most polygons (SW and BIT), where *L. retroversa* predominated (Fig. 10), the estimated biomass values of copepods and pteropods were similar (11 g dw m^−2^, Fig. S16A). The biomass of copepods was higher, and that of pteropods lower (32 and 5 g dw m^−2^, respectively), for the two polygons further into the Barents Sea (HD and TIB, Fig. S16A; note that the high biomass of copepods was strongly influenced by the very high estimated biomass of *C. finmarchicus* at two stations in the HD polygon with ~ 100 g dw m^−2^).

**Fig. 11:**
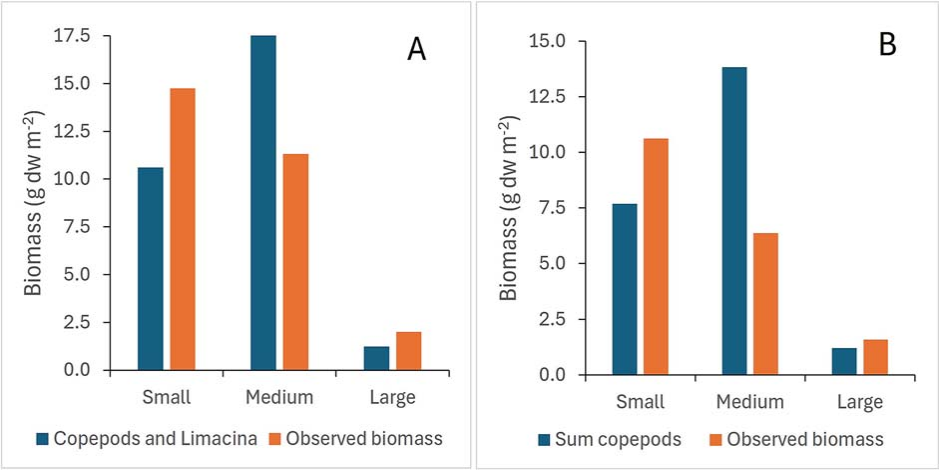
Distribution of estimated biomass (calculated from numbers) as sum for copepods and *Limacina* pteropods (in 1994 only) compared to directly measured biomass of the complementary half-samples for three size fractions in (**A**) 1994 and (**B**) 1995. Average values of biomass across all stations each year (*n* = 23 and 11, respectively).

When the calculated biomass values (from numbers) of copepods and *Limacina* in 1994 were allocated to the three size fractions based on size (width) of taxa, the calculated biomass for the medium size fraction exceeded the directly measured biomass, while the calculated biomass for the small fraction was lower than the measured biomass (Fig. 11A). Most of the calculated biomass for *C. finmarchicus*, dominated by stage C5 (Fig. 9), was ascribed to the medium fraction (80%, Fig. S16B). The calculated biomass for *Limacina* was ascribed mainly to the small fraction due to their small size (7 g dw m^−2^, Fig. S16B).

The samples in 1995 showed similar features as in 1994, with higher estimated biomass of copepods for the medium fraction and lower estimate for the small fraction, compared to the measured biomass (Fig. 11B). The biomass in 1995 was dominated by younger copepodite stages of *C. finmarchicus* (notably C4, Fig. 9, Fig. S14), while small juvenile *Limacina* pteropods occurred with much lower abundance (by an order of magnitude, Table II) and their biomass was not calculated. The estimated biomass of *C. finmarchicus* exceeded the measured total biomass (mean values of 21 versus 19 g dw m^−2^, Table S6), especially for the Atlantic water polygons (SW, BIT and TIB, Fig. S17). The two metrics were positively but not significantly correlated (r = 0.49, p > 0.1). Station 911 in the NE polygon showed a large discrepancy with low estimated biomass of copepods (Fig. S17). Removing this as a potential outlier improved the correlation to r = 0.77 (p ~ 0.01) for the remaining 10 stations.

## DISCUSSION

### Contribution of Limacina pteropods to the biomass peak in 1994

The biomass peak in 1994 was exceptional in two respects—its magnitude, and the relatively high contribution of the small size fraction to total biomass. The mean total biomass in three of the Atlantic polygons was ~ 20 g dw m^−2^ (Fig. S3), with about half of the biomass contained in the small fraction (Table I). This is about twice the long-term average biomass for the Atlantic polygons and differs from the general dominance by the medium fraction, which is driven by *C. finmarchicus* (Skjoldal *et al.*, 2022; Skjoldal and Aarflot, 2023; Skjoldal, 2024; Skjoldal and Sperfeld, 2024). The present results show that the unusual biomass levels in 1994 was due to high abundance of pteropods of *Limacina* species, with a mean value of ~ 0.5 million individuals m^−2^ for the Atlantic polygons, mostly small juveniles < 1 mm in size (Table II, Figs 6 and 7).

The time series data from the Fugløya-Bear Island transect (Fig. 8) strongly suggests a main spawning period for *Limacina* spp. in late summer, in agreement with observations for *L. helicina* in Svalbard fjords (Gannefors *et al.*, 2005; Boissonnot *et al.*, 2021) and suggesting a one-year life cycle. *L. helicina* was reported to have one generation per year at Station Papa in the Gulf of Alaska but was found to have two generations with main spawning in spring and autumn under warmer conditions in an inlet in British Columbia, Canada (Wang *et al.*, 2017). Its Antarctic counterpart, *L. helicina antarctica* or *L. rangii*, has been recorded to have one generation per year with spawning in summer (Hunt *et al.*, 2008; Thibodeau *et al.*, 2020; Gardner *et al.*, 2023). *L. retroversa* has been observed to have two generations per year in the Gulf of Maine, with major spawning activity at the time of the spring bloom and in late summer and autumn (Thabet *et al.*, 2015; Maas *et al.*, 2020).

The two *Limacina* species co-occurred in the Atlantic water mass of the southern Barents Sea. However, we observed a clear difference in that *L. retroversa* predominated in the more recent inflow of Atlantic water in the two southwestern polygons (SW and BIT), while *L. helicina* predominated in the two Atlantic polygons further into the Barents Sea (Figs 1 and 7). This is in line with *L. retroversa* being a boreal species and *L. helicina* being more Arctic. The size frequency distributions in our study suggest active reproduction with high numbers of small juveniles in autumn 1994 (Fig. 6). The size distributions were truncated in the low end with few individuals < 0.2 mm in diameter. This is partly an effect of mesh size, where median (50%) retention occurs for individuals with width ca. equal to the mesh size (~0.2 mm width for the 180-μm WP-2 net; Skjoldal *et al.*, 2013). The smallest individuals collected are presumably veliger larvae (<0.3 mm), which metamorphose into small undifferentiated juveniles <~0.6 mm. The size frequency diagrams in Fig. 6 suggest ongoing recruitment from the small juveniles into identified larger individuals of *L. helicina* in the two polygons furthest into the Barents Sea (HD and TIB), whereas the situation in the BIT polygon suggests that recruitment of *L. retroversa* is nearing the end (indicated by the relatively low abundance of small juveniles).

The Fugløya-Bear Island transect runs S-N across the two westernmost polygons (SW and BIT). The seasonal data from the monitoring at this transect suggests that peak recruitment is in August when the highest abundance of individuals was consistently recorded as a pattern for the 28 years of data (1995–2022, Fig. 8). The species and size of *Limacina* were not determined for this routine monitoring series. If we assume that *L. retroversa* was the predominant species in the southwestern inflow region, as it was in 1994 (Figs 6 and 7), the results suggest that this species has a one-year life cycle at the northern end of its geographical range, with a major spawning in late summer, and with little evidence for spawning in spring as found in warmer environments (e.g. Gulf of Maine, Maas *et al.*, 2020). *L. retroversa* has been observed with peak abundance in the eastern Norwegian Sea in August, when they occurred with high numbers of small juveniles (<0.48 mm) in the surface layer (upper 25 m), with maximum density of 3.3 million individuals m^−2^ (Noji, 1989; Bathmann *et al.*, 1991). By reproducing in late summer, the new generation can develop on late-season growth of phytoplankton, after the dominant copepod *C. finmarchicus* has left the surface layer to spend the winter at depth (Melle *et al.*, 2014).

The strong dominance of small juvenile individuals < 1 mm for both *L. helicina* and *L. retroversa* in autumn 1994 (Fig. 6) is in agreement with results for these two species in fjord waters at Svalbard (Lischka *et al.*, 2011; Boissonnot *et al.*, 2021), for *L. helicina* in coastal waters in the Northeast Pacific (Wang *et al.*, 2017), as well as for the related forms in the Southern Ocean (*L. helicina antarctica* or *L. rangii* and *L. retroversa australis*; Bednaršek *et al.* 2012; Gardner *et al.*, 2023). Thus, results from the Scotia Sea showed a population structure with a marked peak of small juveniles around a modal size ~ 0.3 mm, and with few individuals larger than 1 mm (Bednaršek *et al.*, 2012).

The two species of *Limacina* both appear to have contributed to the high biomass event in 1994, with the largest estimated biomass for *L. retroversa* in the two polygons (SW and BIT) adjacent to the Norwegian Sea (Fig. 10). This suggests that the highly abundant *Limacina* in 1994 was at least partly advected with the Atlantic water from the Norwegian Sea, given the general direction of the currents flowing into the Barents Sea (see Fig. 1; Ingvaldsen *et al.*, 2004, 2021). It appears that conditions were favorable for both species in autumn 1994, but we have no good explanation for what these conditions might have been, nor for the transition between the strong dominance of *L. retroversa* in the two westernmost polygons (SW and BIT) to the dominance of *L. helicina* in the two polygons farther into the Barents Sea (Fig. 7).

Skjoldal *et al.* (2022) examined the time series of size-fractioned zooplankton biomass from the autumn cruises in the Barents Sea from 1989 to 2020 in relation to climate variables and capelin (*Mallotus villosus*) stock size (the Barents Sea capelin is a major predator on zooplankton). A suite of climate variables (temperature of the Atlantic water at the Russian Kola transect (Fig. S2), area (km^2^) of Atlantic and Arctic water masses, and area of winter sea ice cover) were significantly related to the spatio-temporal variation in zooplankton biomass, as was the variation in the capelin stock. Using annual average zooplankton biomass values, “climate” and capelin explained ~ 60% of the interannual variation in zooplankton biomass, with capelin as the strongest factor (inversely related to zooplankton biomass reflecting its role as a major zooplanktivore predator; Skjoldal *et al.*, 2022). On this pattern of explained variance by “climate” and capelin, the year 1994 stood out as a clear outlier in an NMDS plot (non-metric multidimensional scaling; see Fig. 8 in Skjoldal *et al.*, 2022). The main climate signal was a warming trend over the time series (1989–2020), as exemplified by the Kola temperature in Fig. S2 (Boitsov *et al.*, 2012). The year 1994 was intermediate between warm years in the early 1990s (1990–92) and cold years in the late 1990s (1997–1998, Fig. S2). The spring phytoplankton bloom in the Atlantic water of the Barents Sea typically takes place in May (Dalpadado *et al.*, 2020). We have no specific information on any unusual phenology of the spring bloom in 1994 or 1995.

We interpret the high numbers of juveniles observed in Atlantic water in the Barents Sea in autumn 1994 as an exceptional mass occurrence of the *Limacina* species. This is supported by two lines of evidence. First, the counts of gastropods (mainly pteropods) at the Fugløya-Bear Island transect for the following 28 years (1995–2022) generally showed much lower abundance values, with just a few cases up in the range of values observed in 1994 (Fig. 8). Second, the high total zooplankton biomass and the relatively large contribution by the small size fraction for the Atlantic polygons in 1994 differed from the typical situation with strong dominance of the medium fraction and *C. finmarchicus* in the Atlantic water (Aarflot *et al.*, 2018; Skjoldal and Aarflot, 2023; Skjoldal and Sperfeld, 2024). Our values for abundance and biomass for the two *Limacina* species are furthermore high compared to values for *Limacina* species observed in other studies. In a two-years study in the Adventfjord at Svalbard, Boissonnot *et al.* (2021) observed maximum abundance of ~ 100 000 ind. m^−2^ for juvenile *L. helicina* in September. A three-years study in Rivers Inlet in British Columbia (Canada) recorded a maximum abundance of *L. helicina* of ~ 200 000 ind. m^−2^ (Wang *et al.*, 2017). Long-term monitoring in shelf and slope waters in British Columbia and in the eastern Gulf of Alaska found monthly geometric mean abundance values < 5 000 ind. m^−2^ for *L. helicina*, with associated mean biomass values < 0.5 g dw m^−2^ (Mackas and Galbraith, 2012). For the Southern Ocean, Bednaršek *et al.* (2012) reported abundances of *L. helicina antarctica* (= *L. rangii*) across a large dataset from the Scotia Sea ranging from ~ 1 000 to a maximum of nearly 200 000 individuals m^−2^.

### The high biomass in 1995 was driven by *C. finmarchicus*

The high biomass values of ~ 20 g dw m^−2^ in autumn 1994 reappeared at a similarly high level one year later in the BIT polygon (Fig. S3). This gave an impression of a lingering feature which could have been a continuation of the mass occurrence of *Limacina* spp. Analysis of samples from 1995 showed that this was not the case. The abundance of *Limacina* in 1995 was an order of magnitude lower than in 1994 (Fig. 5A, Table II). Instead, *C. finmarchicus* occurred with exceptionally high abundance and with dominance of young copepodite stages (C4 and C3; Fig. 5B). The typical situation for *C. finmarchicus* in Atlantic water in autumn is to occur with a dominance of stage C5 but also with some stage C4 (Aarflot *et al.*, 2018). This was seen at the Fugløya-Bear Island transect for samples collected in August 1995–2019 (Skjoldal *et al.*, 2021). An exception was a sample from an Atlantic water station (at 73^o^N) in August 1995, with high abundance (~0.6 million ind. m^−2^) and dominance of young copepodite stages (Skjoldal *et al.*, 2021). Our new analyses of samples from 1995 show that the feature of high abundance with dominance of young stages (C2–C4) occurred widespread in Atlantic polygons (SW, BIT and TIB) in autumn 1995 (Fig. S10). The maximum recorded abundance of 1.5 million *Calanus* copepodites m^−2^ in 1995 (at station 824 in BIT, Fig. S10) is the record for samples analyzed from the Barents Sea.

*C. finmarchicus* also contributed to the high biomass values in 1994, in this case dominated by the overwintering stage C5 (Fig. 9, Fig. S13). The mean biomass of *C. finmarchicus* (calculated from abundance) was relatively high (21 g dw m^−2^, Fig. 9). Thus, the situation in 1994 appeared to be one where *C. finmarchicus* was present in typical abundance, with the mass occurrence of *Limacina* pteropods representing an additional biomass on top of that of *C. finmarchicus*. The mean abundance of *C. finmarchicus* for the 23 stations in 1994 was ~ 140 000 copepodites m^−2^ (Table II), which is somewhat higher than mean values of 70–90 thousand for two Atlantic water stations at the Fugløya-Bear Island transect during 25 years of sampling (1995–2019, Skjoldal *et al.*, 2021). However, the biomass for the 23 stations with species counts was also higher than the general average biomass for the four Atlantic polygons in 1994 (28 vs 18 g dw m^−2^, Table I and Table S6). This reflects the fact that the stations for species counts were selected from the high end of the biomass values, since our primary objective was to establish what drove these high biomass values in 1994.

### Measured and estimated biomass in size fractions

The size-fractioned biomass for the Atlantic polygons showed a dominance of the small size fraction (~50%) over the medium fraction (~40%) in both 1994 and 1995 (Fig. 3, Table I). The high contribution of the small fraction occurred across the range of biomass values with dominance also at high total zooplankton biomass (Fig. 4). This differs from the general pattern of lower contribution of the small fraction at high biomass levels (Skjoldal and Sperfeld, 2024), like the pattern we found for the “other” polygons in 1994 (Fig. 4). Skjoldal and Sperfeld (2024) used data for the period 1989–2020 and noted a deviating pattern for 1994 and 1995. We can now explain the deviating pattern as due to high abundances of small juvenile *Limacina* pteropods in 1994 and of young copepodites of *C. finmarchicus* in 1995, both contributing to high biomass of the small fraction as well as total zooplankton biomass.

The estimated biomass of copepods (mainly *C. finmarchicus*, Fig. S12) and *Limacina* spp. (Fig. 10), calculated from numbers and individual size (weight), was in good agreement with the directly measured biomass in the complementary half-samples (Figs S15 and S17). The two metrics (measured and calculated biomass) were strongly correlated across the 23 stations in 1994 (r = 0.87). Removing one potential outlier from the dataset for 1995 improved the correlation to become statistically significant (r = 0.77, *n* = 10, *P* ~ 0.01).

The results for 1995 suggest that the biomass of *C. finmarchicus* was overestimated. Thus, the estimated biomass of copepods exceeded the measured total biomass for all but one station in the Atlantic polygons (Fig. S17) and was on average higher by 22% across all stations (Table S6). We used weights reduced by 30% for *C. finmarchicus* copepodite stages compared to values used by Aarflot *et al.* (2018). A rationale for this was the likely occurrence of a second (summer, G2) generation of *C. finmarchicus* (Skjoldal *et al.*, 2021), with smaller individuals compared to the spring G1 generation (Skjoldal and Aarflot, 2023). It is likely that the population in 1995 with high abundance of young copepodites reflected a delayed development of the G2 summer generation (Skjoldal *et al.*, 2021), made up of smaller individuals than usual. The weights of *C. finmarchicus* copepodites used by Aarflot *et al.* (2018) were based on a literature review. The weight of stage C5 was 250 μg dw, reduced to 175 μg dw (30% reduction) in our case. A further reduction to e.g. 50% (125 μg dw for C5, and corresponding reductions for other copepodite stages) might be appropriate for the situation in 1995, although we have not pursued this further.

The estimated combined biomass of copepods and *Limacina* pteropods in 1994 was comparable to the directly measured total zooplankton biomass (Table S6). Due to uncertainties in the estimates of biomass for both *C. finmarchicus* and *Limacina* spp., it is difficult to evaluate the precision of the combined estimates, e.g. whether the biomass of *C. finmarchicus* was overestimated also in 1994 when the population structure was different from 1995 (Fig. 5B).

The biomass of the *Limacina* species was calculated as that of a sphere (2/3 of a sphere for the more compressed *L. helicina*) assuming a density of 1.0 (g cm^−3^). We chose this option because there were considerable discrepancies in reported relationships (power function) between weight and diameter for the two *Limacina* species (see Method, section *Estimation of biomass of copepods and* Limacina *pteropods,* and Fig. S5; Conover and Lalli, 1972; Gannefors *et al.*, 2005; Bednaršek *et al.*, 2012). One reason for the discrepancy is probably inaccuracies in the low end of the regressions influenced by large variation in measured weights of larger individuals. The small juvenile individuals are globular in shape, and we believe a sphere is a reasonable approximation of volume. Volume was converted to dry weight by factor 0.2, corresponding to 80% water content, which is close to what has been found for the *Limacina* species (Ikeda and Skjoldal, 1989; Gannefors *et al.*, 2005; Ikeda, 2014). The ash content of pteropod biomass (due to the shell) is typically around 30–40% (Omori, 1969; Ikeda and Skjoldal, 1989; Gannefors *et al.*, 2005), which is somewhat higher than for copepod biomass (~10%, Kiørboe, 2013).

The calculated biomass of copepods was allocated to the three size fractions based on results in Skjoldal (2021), where copepods shifted progressively from the small to the medium fraction (being retained on the 1-mm screen) as their width increased from 0.4 to 0.8 mm. This is interpreted to reflect that copepods pass the sieving-screen as a combined function of their length and width (length of copepods is typically ~ 3 times the width; Pearre Jr., 1980). With the more globular shaped *Limacina* pteropods, the distinction between width and length disappears. We therefore used a cut-off limit at 1 mm diameter (same as the 1-mm screen separating the small and medium fractions), ascribing individuals < 1 mm to the small fraction and individuals > 1 mm to the medium fraction. This approximates the logistic function for retention in a zooplankton net, where 50% retention occurs at width equal to mesh size (Skjoldal *et al.*, 2013).

Compared to the directly measured biomass, the biomass estimated from numbers was lower for the small size fraction and higher for the medium fraction in both 1994 and 1995 (Fig. 11). In 1995, the estimated biomass was for copepods, and smaller individuals of *C. finmarchicus* was possibly the reason why the estimated biomass distribution between the small and medium fractions differed from the measured biomass. Copepodite stage C4 (which was the predominant stage in 1995; Fig. 5B) is split ~ 50:50 between the two fractions, and this split is sensitive to the size of the individuals (Skjoldal, 2021). If the size we used to estimate biomass was too large, the biomass in the small and medium fractions would be under- and overestimated, respectively. In 1994, the calculated biomass of copepods was strongly dominated by stage C5 of *C. finmarchicus* (Fig. 9). Any overestimation of their size would mean little for the splitting into small and medium fractions, since the smaller individuals of stage C5 would still be large enough to be retained and found mainly in the medium fraction (Skjoldal, 2021). However, the higher estimated biomass for the medium fraction in 1994 suggests that the individuals of *C. finmarchicus* were smaller and of lower weight than what we used in the calculation. *Limacina* made up nearly 2/3 of the calculated biomass of the small size fraction in 1994 (Table S6). The fact that the calculated biomass was lower than the measured biomass for the small fraction this year suggests that we may have underestimated the calculated biomass of the small juvenile *Limacina* (Fig. S16B).

### The 1994 biomass peak in perspective

The biomass of the 1994 peak in Atlantic water polygons of ~ 20 g dw m^−2^ is exceptional in context of the more than 40 years of observations from research and monitoring in the Barents Sea (Skjoldal, 2024). In the central Barents Sea, including the HD polygon, there were three clear peaks in biomass in 1980, 1987 and 1994. The peak in 1980 was recorded as displacement volume, and this brings some uncertainty regarding its comparability with subsequent peaks recorded with the standard method of size-fractioned dry weight biomass (Skjoldal, 2024). The peaks in 1987 and 1994 occurred when the Barents Sea capelin stock was at low levels, and they have been part of the pattern of an inverse relationship between zooplankton biomass and the size of the capelin stock (Stige *et al.*, 2014; Dalpadado *et al.*, 2020). Dalpadado *et al.* (2003) noted a difference between the 1987 and 1994 peaks in that the former occurred in Arctic water and the latter in Atlantic water. The 1994 peak occurred mostly upstream of the core capelin feeding area and was likely an advective feature of the Atlantic inflow rather than a response to low capelin predation (Skjoldal *et al.*, 2022). The present results showing exceptional numbers of the boreal pteropod *L. retroversa* (Fig. 7) support this interpretation. Removing data for 1994 weakened the inverse relationship between zooplankton biomass and capelin stock size (Dalpadado *et al.*, 2020), but the inverse relationship remained significant when calculated for the central core capelin feeding area (Skjoldal *et al.*, 2022).

The 1994 peak in zooplankton biomass in the southern Barents Sea coincided with a pattern of exceptional changes called “regime-shift”-like that took place in the Northeast Atlantic in the mid-1990s, related to large-scale climate forcing (Alheit *et al.*, 2019). In the adjacent Norwegian Sea, Atlantic herring (*Clupea harengus*) of the recovering Norwegian spring-spawning stock had started to return to the traditional summer feeding grounds following the stock collapse in the late 1960s (Holst *et al.*, 2004). The NAO index remained in a high positive state from 1988 to 1995, before a strong drop to negative mode in 1996 (Skjoldal and Sætre, 2004). Between 1995 and 1997, the zooplankton biomass in the Norwegian Sea in spring (May) showed a pronounced drop coinciding with a decrease in body condition of herring (Holst *et al.*, 2004). However, any links between what happened in the Norwegian Sea in the mid-1990s and the exceptional mass occurrence of *Limacina* pteropods in the Atlantic inflow to the Barents Sea in 1994 remain to be investigated retrospectively.

## CONCLUSION

The peak in zooplankton biomass in the Barents Sea in 1994, seen from time series monitoring, was driven by mass occurrence of small juveniles (<1 mm) of thecosome pteropods *Limacina* species. The boreal *L. retroversa* and the Arctic *L. helicina* both contributed to the high biomass event but with *L. retroversa* as the most important of the two species. The high biomass event took place mainly in Atlantic water in the southwestern inflow region to the Barents Sea and is interpreted to be an advective phenomenon from the adjacent Norwegian Sea. The high biomass values reflected an additional effect of the *Limacina* pteropods on top of a typical abundance and biomass of a second generation (G2) of *C. finmarchicus*. Similarly high biomass values in parts of the Atlantic inflow region the following year (1995) had a different explanation and was due to high abundance of a delayed summer generation of *C. finmarchicus* with dominance of younger and smaller copepodite stages.

The two *Limacina* species both appear to have a one-year life cycle in the Atlantic water mass of the Barents Sea, with a major spawning event in late summer. The retrospective analysis showed that the similarity in the unusual biomass composition in 1994 and 1995 (high biomass of the small size fraction and total) was only apparent, driven by small *Limacina* pteropods in 1994 and a delayed summer generation of *C. finmarchicus* dominated by young copepodite stages in 1995. The results for 1994 and 1995 provide context that contributes to a better understanding of the Barents Sea as well as other boreal and sub-arctic ecosystems.

## Supplementary Material

Supplementary_material_rev_080225_fbaf012

Supplementary_Tables_S4_and_S5_fbaf012

## Data Availability

The data are stored in the database at the Institute of Marine Research in Norway, Norwegian Marine Data Centre.
