# Supplementary material for: A mass occurrence of pteropods (Limacina spp.) drove a pronounced peak in zooplankton biomass in Atlantic water in the Barents Sea in 1994
Source: J Plankton Res. 2025 Mar 27;47(2):fbaf012. doi: 10.1093/plankt/fbaf012 (PMC11950534; doi:10.1093/plankt/fbaf012)
Supplement: Supplementary_material_rev_080225_fbaf012 [file supplementary_material_rev_080225_fbaf012.docx]

***A mass occurrence of pteropods (*Limacina *spp.) drove a pronounced peak in zooplankton biomass in Atlantic water in the Barents Sea in 1994***

Hein Rune Skjoldal, Espen Bagøien and Monica Bente Martinussen

**Supplementary material**

Table S-1. List of sampling stations in 1994 and 1995 with zooplankton samples analyzed for taxonomic composition.

| Year/Ship | Station # | Polygon | Latitude | Longitude | Depth (m) | Date |
| --- | --- | --- | --- | --- | --- | --- |
| 1994 |  |  |  |  |  |  |
| G.O. Sars | 1166 | SW | 70.50 | 20.00 | 131 | 3-Oct |
| G.O. Sars | 1160 | SW | 71.50 | 19.80 | 235 | 3-Oct |
| Johan Hjort | 765 | SW | 71.49 | 22.99 | 419 | 3-Sep |
| G.O. Sars | 1006 | SW | 71.53 | 25.75 | 291 | 2-Sep |
| G.O. Sars | 975 | SW | 70.75 | 31.22 | 287 | 25-Aug |
| G.O. Sars | 980 | SW | 72.00 | 31.22 | 336 | 27-Aug |
| G.O. Sars | 1027 | BIT | 72.50 | 17.30 | 365 | 6-Sep |
| G.O. Sars | 1146 | BIT | 72.33 | 20.00 | 337 | 1-Oct |
| G.O. Sars | 1156 | BIT | 72.50 | 19.57 | 393 | 2-Oct |
| G.O. Sars | 1140 | BIT | 72.83 | 20.00 | 427 | 30-Sep |
| G.O. Sars | 1139 | BIT | 72.83 | 22.00 | 424 | 30-Sep |
| G.O. Sars | 1154 | BIT | 73.00 | 19.47 | 419 | 2-Oct |
| G.O. Sars | 1116 | HD | 74.33 | 26.00 | 400 | 25-Sep |
| G.O. Sars | 1118 | HD | 74.33 | 30.00 | 340 | 25-Sep |
| G.O. Sars | 989 | HD | 74.25 | 31.22 | 290 | 28-Aug |
| G.O. Sars | 1104 | HD | 74.83 | 28.00 | 356 | 22-Sep |
| G.O. Sars | 996 | HD | 75.00 | 28.75 | 361 | 30-Aug |
| G.O. Sars | 1096 | HD | 75.33 | 32.00 | 320 | 21-Sep |
| G.O. Sars | 992 | HD | 75.50 | 31.22 | 363 | 29-Aug |
| G.O. Sars | 982 | TIB | 72.50 | 31.22 | 304 | 27-Aug |
| G.O. Sars | 987 | TIB | 73.75 | 31.22 | 362 | 28-Aug |
| G.O. Sars | 1122 | TIB | 73.83 | 32.00 | 347 | 26-Sep |
| G.O. Sars | 1121 | TIB | 73.77 | 34.05 | 314 | 26-Sep |
| 1995 |  |  |  |  |  |  |
| Johan Hjort | 1193 | SW | 71.24 | 30.97 | 270 | 8-Oct |
| G.O. Sars | 842 | SW | 71.30 | 20.94 | 269 | 31-Aug |
| G.O. Sars | 847 | SW | 71.88 | 23.96 | 297 | 1-Sep |
| G.O. Sars | 824 | BIT | 73.00 | 19.47 | 413 | 28-Aug |
| Johan Hjort | 1057 | BIT | 73.11 | 23.28 | 391 | 30-Aug |
| G.O. Sars | 882 | TIB | 72.50 | 31.22 | 298 | 9-Sep |
| Johan Hjort | 1064 | TIB | 72.57 | 28.30 | 341 | 4-Sep |
| G.O. Sars | 942 | NE | 73.25 | 50.00 | 259 | 23-Sep |
| G.O. Sars | 911 | NE | 78.00 | 47.50 | 331 | 18-Sep |
| G.O. Sars | 914 | NE | 76.50 | 47.50 | 190 | 18-Sep |
| G.O. Sars | 917 | NE | 76.50 | 53.50 | 173 | 18-Sep |

Table S-2. Two-ways ANOVA with interaction term for the effects of years (1994 and 1995) and geographical areas/water masses (Atlantic versus ‘other’ polygons, see Fig. S-1) on zooplankton biomass (g dw m^-2^) for three size fractions (small <1 mm, medium 1-2 mm, large >2 mm) and total. Significance is shown as: * p <0.05, ** p <0.01, *** p <0.001. d.f. – degrees of freedom.

| Size fraction | Factor | Sum Sq. | d.f. | F-value | p-value | Significance |
| --- | --- | --- | --- | --- | --- | --- |
|  |  |  |  |  |  |  |
| Small | Intercept | 1.5813 | 1 | 18.48 | <0.001 | *** |
|  | Year | 1.576 | 1 | 18.42 | <0.001 | *** |
|  | Polygon | 1.068 | 1 | 12.48 | <0.001 | *** |
|  | Year x Polygon | 1.067 | 1 | 12.47 | <0.001 | *** |
|  | Residuals | 24.64 | 288 |  |  |  |
|  |  |  |  |  |  |  |
| Medium | Intercept | 0.7970 | 1 | 6.43 | 0.012 | * |
|  | Year | 0.794 | 1 | 6.41 | 0.012 | * |
|  | Polygon | 0.664 | 1 | 5.36 | 0.021 | * |
|  | Year x Polygon | 0.664 | 1 | 5.35 | 0.021 | * |
|  | Residuals | 35.69 | 288 |  |  |  |
|  |  |  |  |  |  |  |
| Large | Intercept | 9.26 | 1 | 20.24 | <0.001 | *** |
|  | Year | 9.27 | 1 | 20.25 | <0.001 | *** |
|  | Polygon | 4.76 | 1 | 10.40 | 0.001 | ** |
|  | Year x Polygon | 4.76 | 1 | 10.41 | 0.001 | ** |
|  | Residuals | 131.83 | 288 |  |  |  |
|  |  |  |  |  |  |  |
| Total | Intercept | 1.2009 | 1 | 16.81 | <0.001 | *** |
|  | Year | 1.195 | 1 | 16.72 | <0.001 | *** |
|  | Polygon | 0.927 | 1 | 12.97 | <0.001 | *** |
|  | Year x Polygon | 0.926 | 1 | 12.96 | <0.001 | *** |
|  | Residuals | 20.57 | 288 |  |  |  |

Table S-3. Regression equations for linear relationships between the small, medium, and large size fractions versus total zooplankton biomass for Atlantic and ‘other’ polygons in 1994 and 1995 (see Fig. 4). Regression slope is given with standard error (SE), t value, and probability for slope being different from 0 (zero). R^2^ (adjusted) is fraction of variance explained by the linear regression.

| Data | | Fraction | n | Intercept | Slope | SE (slope) | t | p | R^2^ (adj) |
| --- | --- | --- | --- | --- | --- | --- | --- | --- | --- |
|  |  |  |  |  |  |  |  |  |  |
| 1994 | Atlantic | Small | 66 | -0.241 | 0.952 | 0.074 | 12.9 | <0.001 | 0.72 |
|  |  | Medium | 66 | -0.571 | 1.087 | 0.089 | 12.3 | <0.001 | 0.70 |
|  |  | Large | 66 | -1.452 | 1.191 | 0.301 | 4 | <0.001 | 0.18 |
|  | Other | Small | 109 | -0.225 | 0.724 | 0.067 | 10.8 | <0.001 | 0.52 |
|  |  | Medium | 109 | -0.557 | 1.207 | 0.062 | 19.4 | <0.001 | 0.78 |
|  |  | Large | 109 | -1.480 | 1.538 | 0.24 | 6.4 | <0.001 | 0.27 |
|  |  |  |  |  |  |  |  |  |  |
| 1995 | Atlantic | Small | 51 | -0.27 | 0.936 | 0.094 | 10 | <0.001 | 0.66 |
|  |  | Medium | 51 | -0.39 | 0.951 | 0.119 | 8 | <0.001 | 0.56 |
|  |  | Large | 51 | -2.76 | 2.043 | 0.522 | 3.9 | <0.001 | 0.22 |
|  | Other | Small | 66 | -0.41 | 0.916 | 0.096 | 9.5 | <0.001 | 0.58 |
|  |  | Medium | 66 | -0.41 | 1.04 | 0.099 | 10.5 | <0.001 | 0.63 |
|  |  | Large | 66 | -1.25 | 1.161 | 0.478 | 2.4 | <0.05 | 0.07 |

Table S-4. Numerical abundance (number of individuals m^-2^) for 23 sampling stations from four Atlantic polygons (SW, BIT, HD and TIB) in 1994.

The table is included in Excel fil ‘Supplementary Tables S-3 and S-4’.

Table S-5. Numerical abundance (number of individuals m^-2^) for 11 sampling stations from three Atlantic polygons (SW, BIT, and TIB) and the NE polygon in 1995.

The table is included in Excel fil ‘Supplementary Tables S-3 and S-4’.

Table S-6. Estimated biomass (calculated from numbers) of copepods in 1994 and 1995 and of pteropods (*Limacina* species) in 1994 in three size fractions (small <1 mm, medium 1-2 mm, large >2 mm) and total (sum of fractions). Mean values for stations in each year (n = 23 and 11 in 1994 and 1995, respectively) compared to mean measured biomass (with standard deviation, SD) for the same sets of stations.

| Year | Taxon | Biomass (g dry weight m^-2^) | | |  |
| --- | --- | --- | --- | --- | --- |
|  |  | Small | Medium | Large | Sum |
| 1994 | *Calanus finmarchicus* | 3.40 | 16.44 | 1.04 | 20.88 |
|  | Other copepods | 0.64 | 0.47 | 0.18 | 1.29 |
|  | *Limacina* spp. | 2.10 | 0.00 |  | 2.10 |
|  | *L. retroversa* | 1.04 | 0.51 |  | 1.55 |
|  | *L. helicina* | 3.43 | 0.22 |  | 3.65 |
|  | Sum copepods and *Limacina* | 10.60 | 17.64 | 1.22 | 29.46 |
|  | Measured biomass | 14.75 | 11.31 | 2.01 | 28.06 |
|  | SD | 8.08 | 14.28 | 2.61 | 22.12 |
|  |  |  |  |  |  |
| 1995 | *Calanus finmarchicus* | 6.57 | 13.00 | 1.01 | 20.58 |
|  | Other copepods | 1.11 | 0.83 | 0.19 | 2.12 |
|  | Sum copepods | 7.68 | 13.83 | 1.19 | 22.70 |
|  | Measured biomass | 10.62 | 6.37 | 1.57 | 18.56 |
|  | SD | 7.27 | 4.50 | 2.09 | 6.54 |


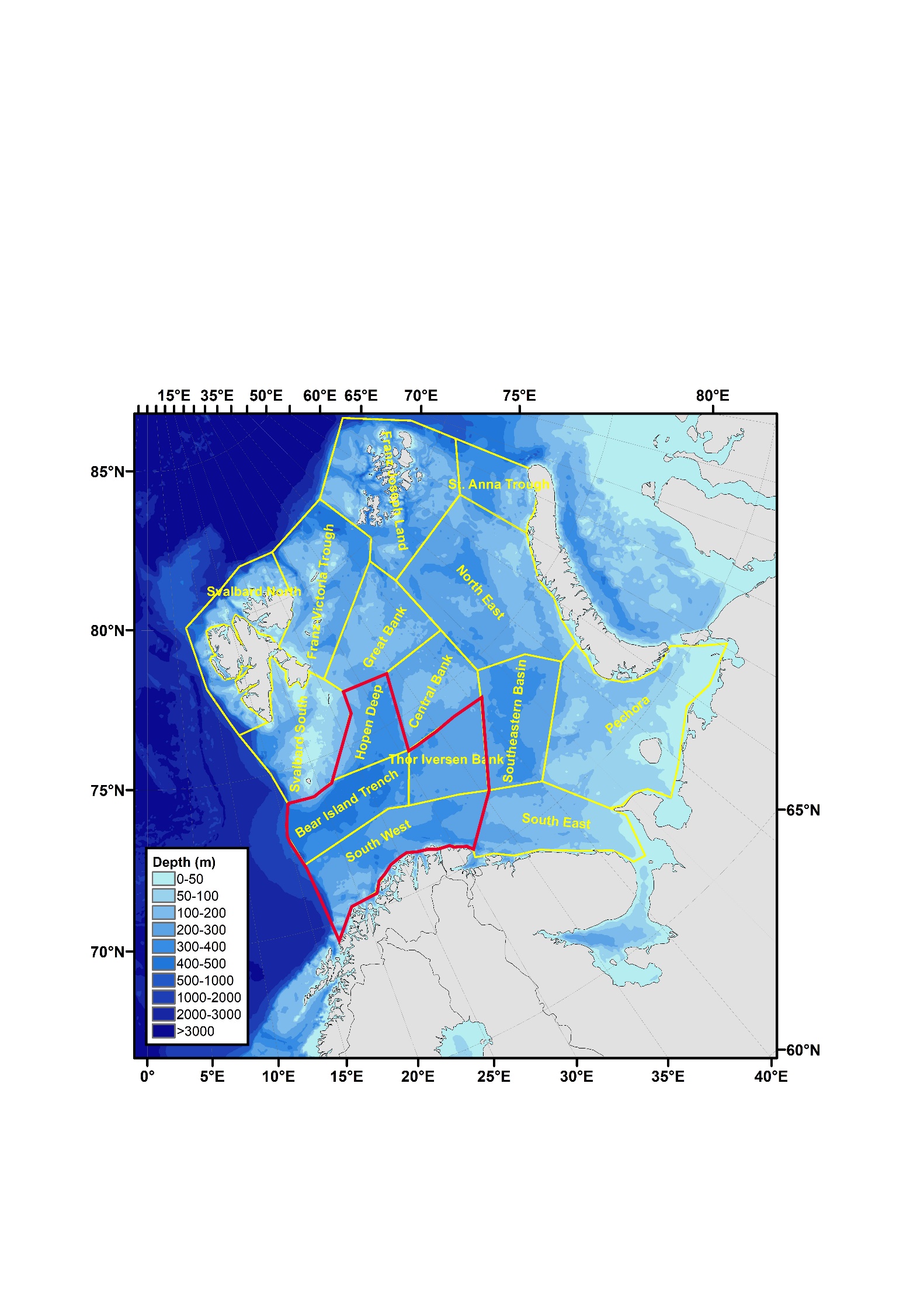


Fig. S-1. Map of the Barents Sea with subdivision into 15 subareas or polygons. The inflow of Atlantic water takes place in the South-West and Bear Island Trench polygons and continues east through the Thor Iversen Bank polygon and north into the Hopen Deep polygon (see Fig. 1 in the main paper). These 4 polygons are denoted as the Atlantic water polygons shown by red lines.


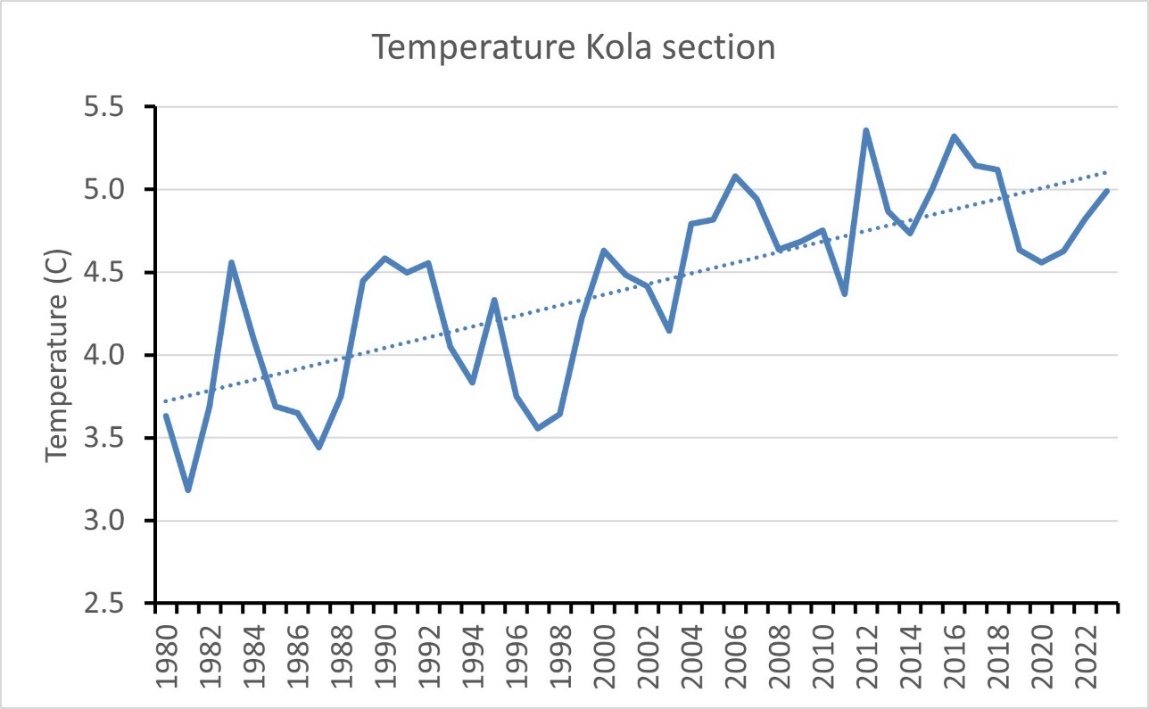


Fig. S-2. Temperature of Atlantic water (0-200 m) at the Russian Kola transect along 33.5^o^E longitude in the southeastern Barents Sea. Annual mean values for 1980-2023 based on monthly values. Updated from Boitsov et al. (2012).


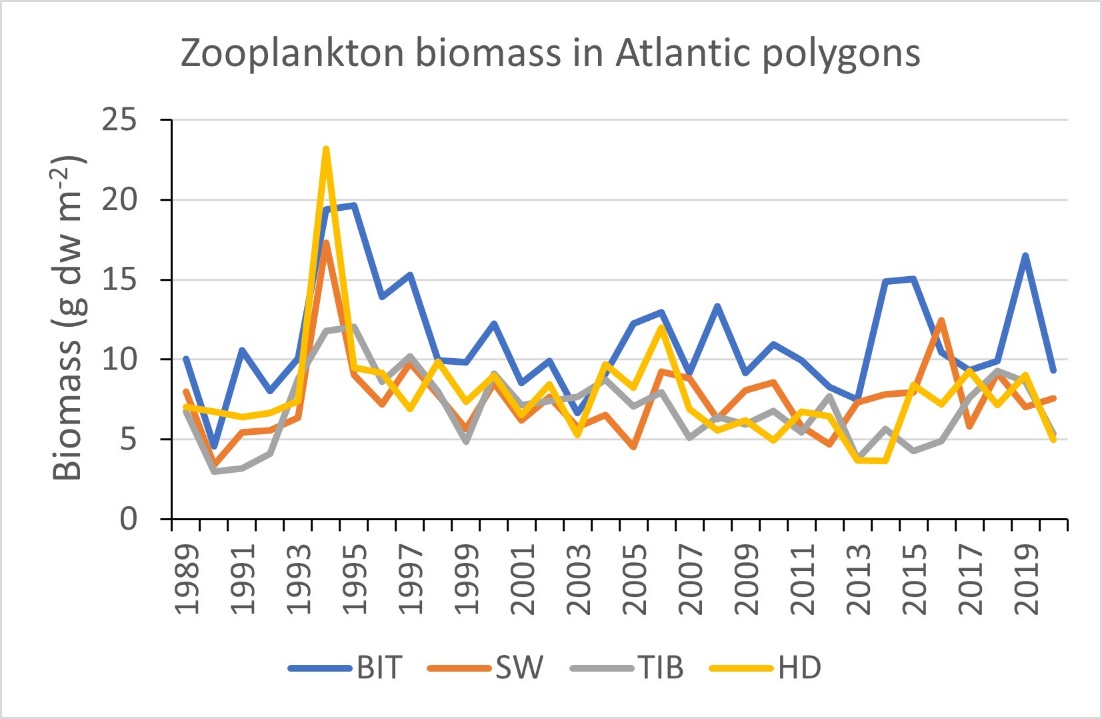


Fig. S-3. Time series of zooplankton biomass (dry weight) for four Atlantic water polygons in the Barents Sea (see map in Fig. S-1) from 1989 to 2020. BIT – Bear Island Trench, SW – South-West, TIB – Thor Iversen Bank, HD – Hopen Deep. See Skjoldal et al. (2022) for more details.


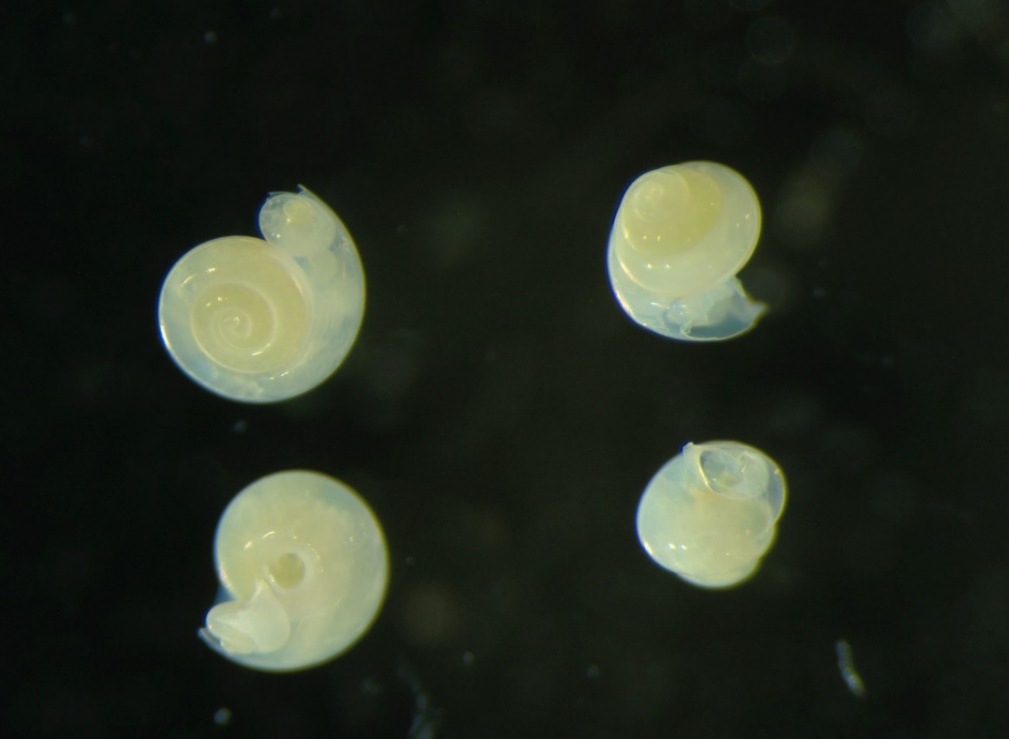


Fig. S-4. Photos of *Limacina helicina* (left) and *L. retroversa* (right) with dorsal (upper) and ventral (lower) views. *L. helicina* is more compressed compared to *L. retroversa*. Note that a smaller juvenile individual is seen in the shell opening of the photo of *L. helicina*. The size (diameter) is 0.63 mm for *L. retroversa* and 0.69 mm for *L. helicina*.


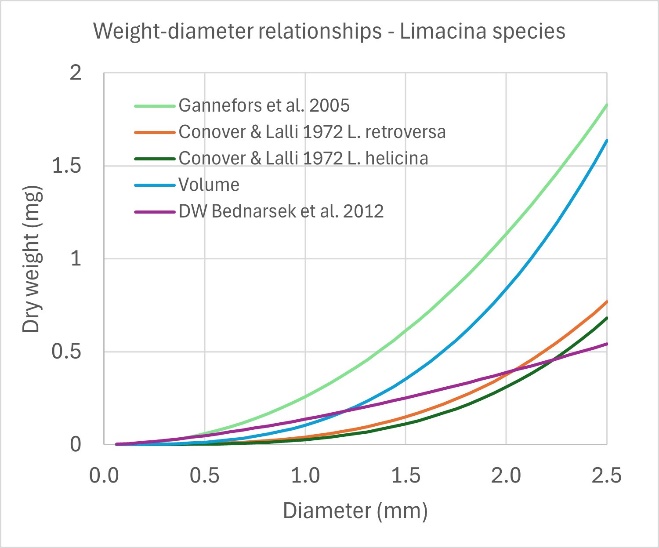

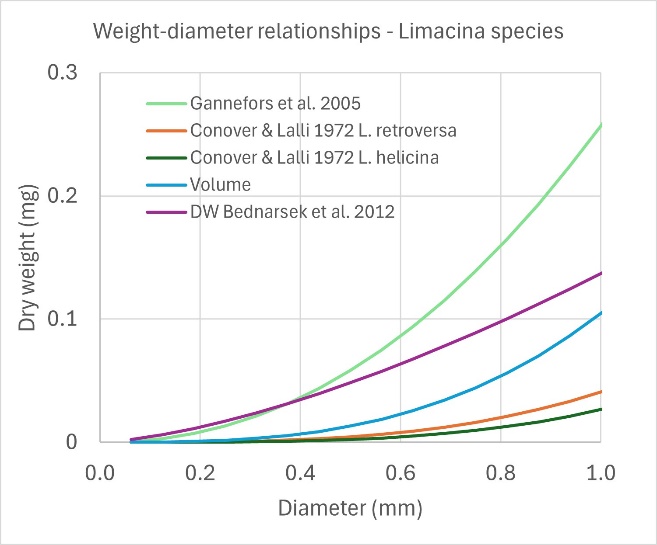


Fig. S-5. Relationships between individual dry weight (W, mg) and diameter (D, mm) of *Limacina* pteropods. Published power function relationships: Gannefors et al. (2005) for *Limacina helicina* (W = 0.257 D^2.141^), Conover and Lalli (1972) for *L. retroversa* (W = 0.0408 D^3.2035^) and *L. helicina* (W = 0.0266 D^3.5387^) (Conover and Lalli 1972 recorded weight as ash-free dry weight), and Bednarsek et al. (2012) for *L. helicina antarctica* (W = 0.137 D^1.5005^). The curve ‘Volume’ is volume for a sphere converted to dry weight by assuming 20 % of volume. The two panels are the same, but with scales zoomed-in for diameter <1 mm in the right panel.


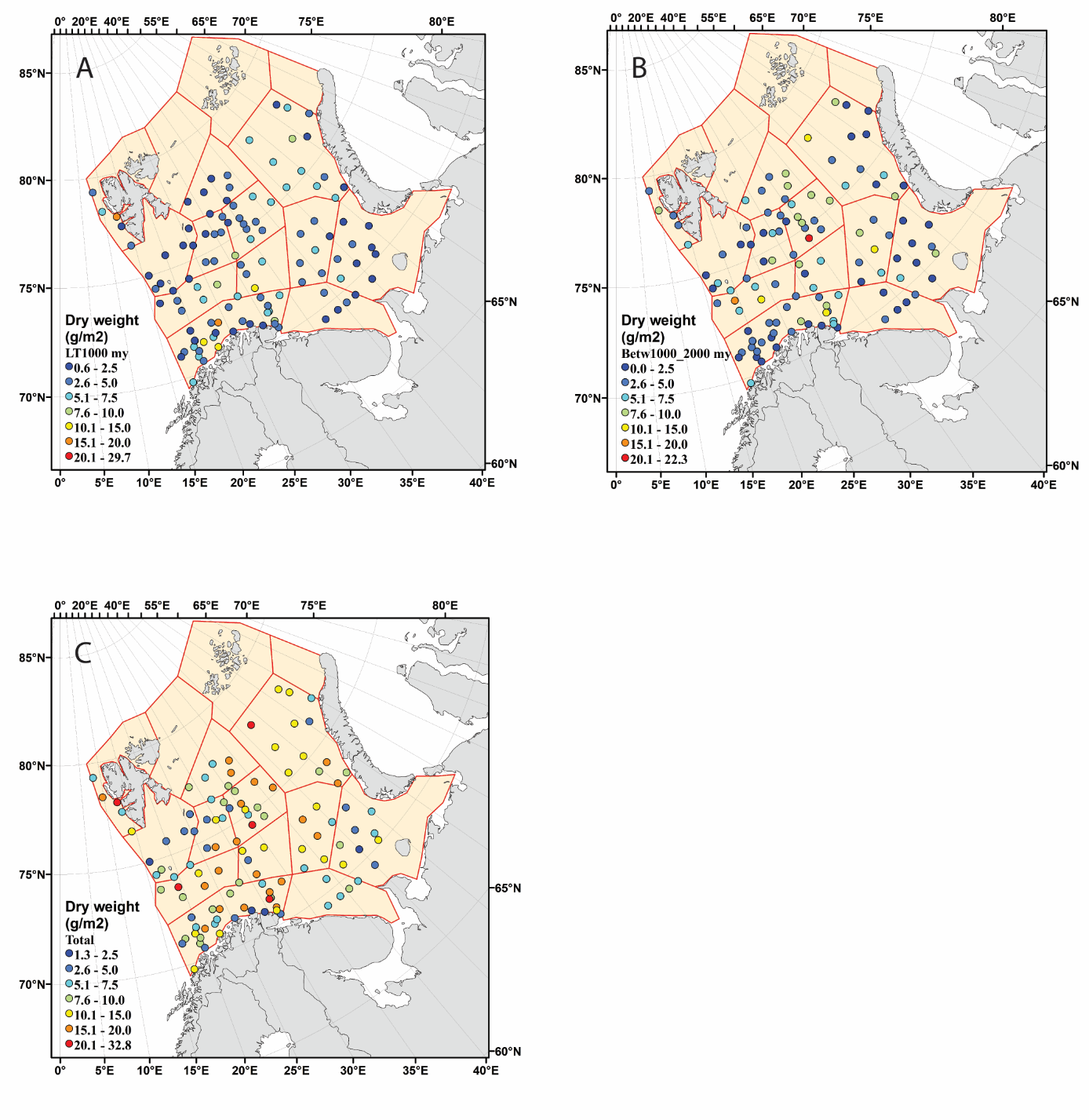


Fig. S-6. Distribution of zooplankton biomass (g dw m^-2^) in the Barents Sea in autumn 1995 for (A) the small (< 1 mm) and (B) medium (1-2 mm) size fractions, and (C) total biomass.


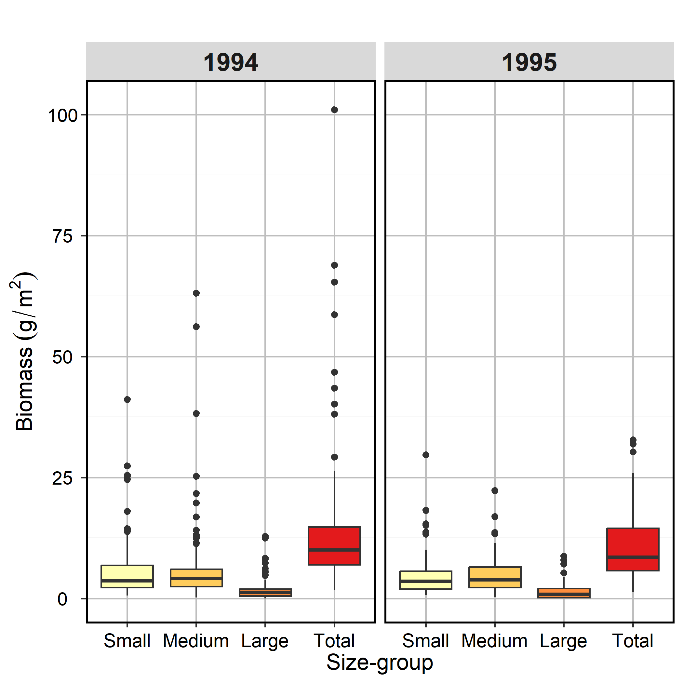

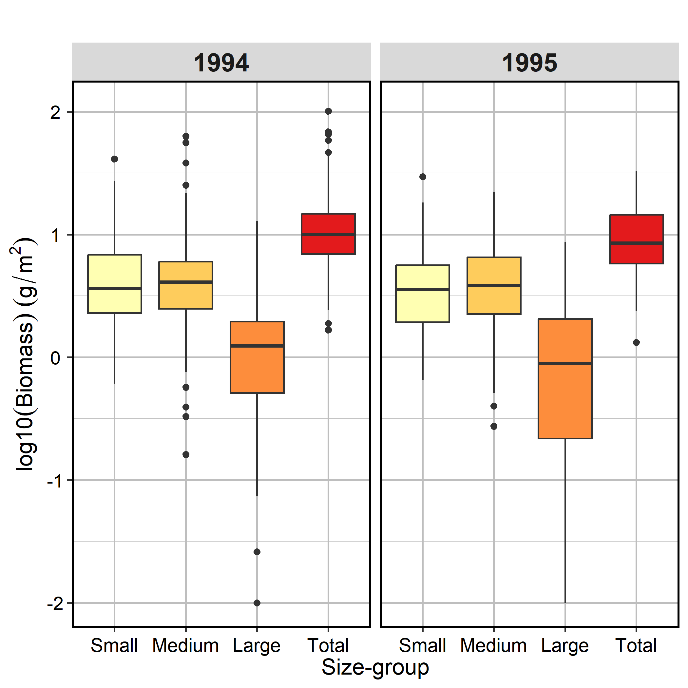


Fig. S-7. Box-whisker plots of zooplankton biomass in three size fractions and total (sum of fractions) for samples collected in the autumn surveys in 1994 (n = 175 stations) and 1995 (n = 117 stations) (see maps in Fig. 2 and Fig. S-6). The same data are shown on linear scale for the plots on the left side, and on log10 scale for the plots on the right side. The plots show median value (horizontal line), 25-75 % quantiles (box), and range of data (vertical line) apart from observations defined as outliers (dots).


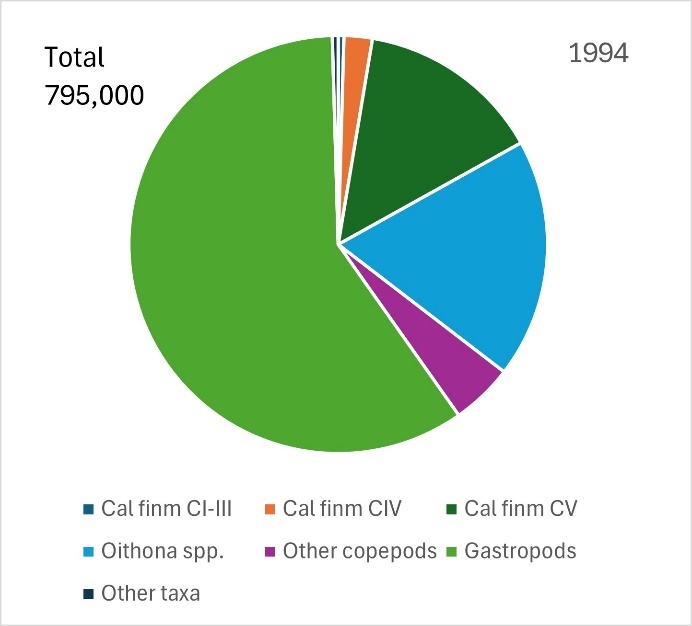

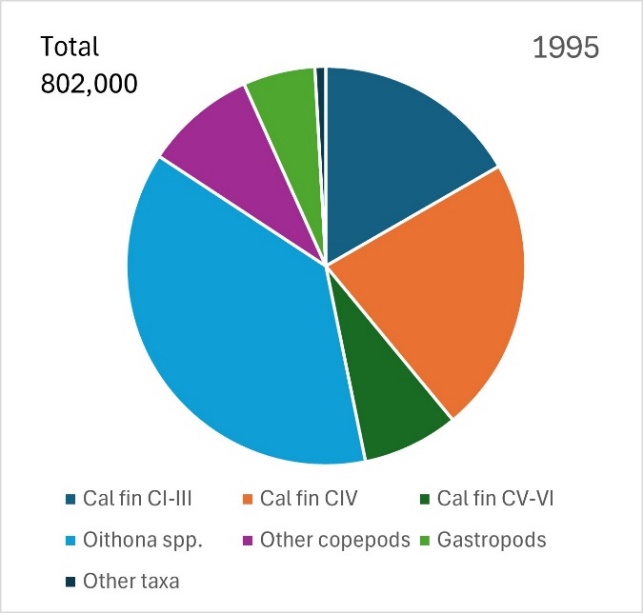


Fig. S-8. Average zooplankton composition by species and groups for the samples from 1994 (left panel, n = 23 stations) and 1995 (right panel, n = 11 stations). Values for *Calanus finmarchicus* are shown separately for copepodite stages CI-III, CIV, and CV-CVI.


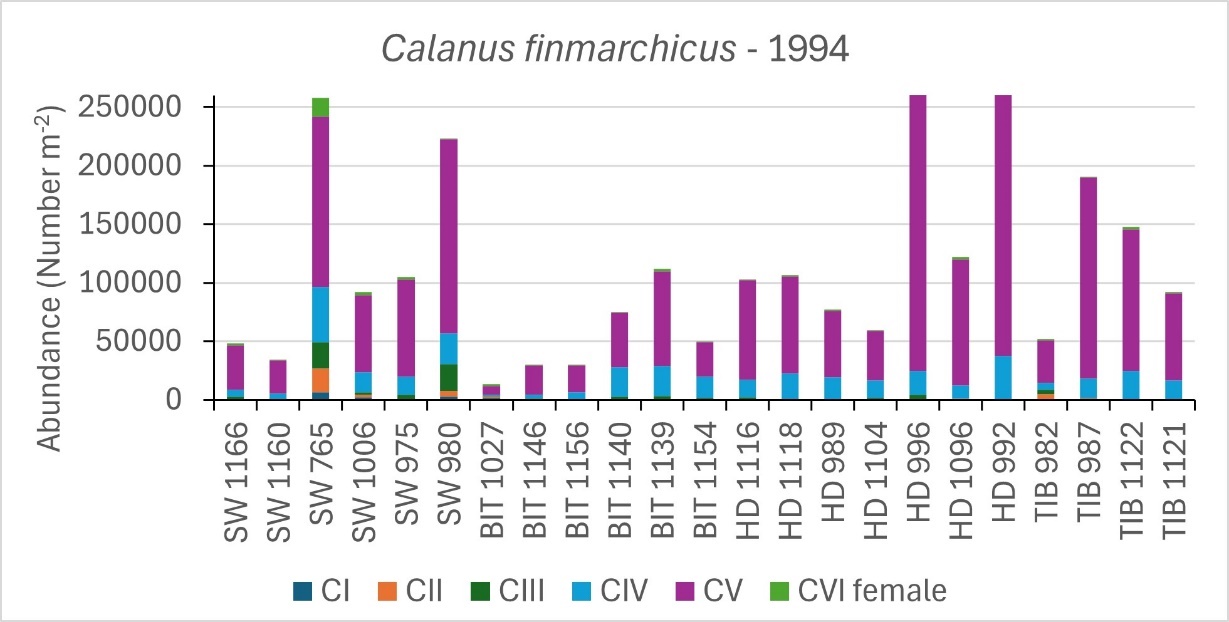


Fig. S-9. Copepodite stage composition of *Calanus finmarchicus* at stations in four Atlantic polygons (SW, BIT, HD, TIB) in 1994. The scale has been cut at 260,000 individuals m^-2^ and does not show the maximum values of 557,000 ind. m^-2^ at station HD 996 and 607,000 ind. m^-2^ at station HD 992.


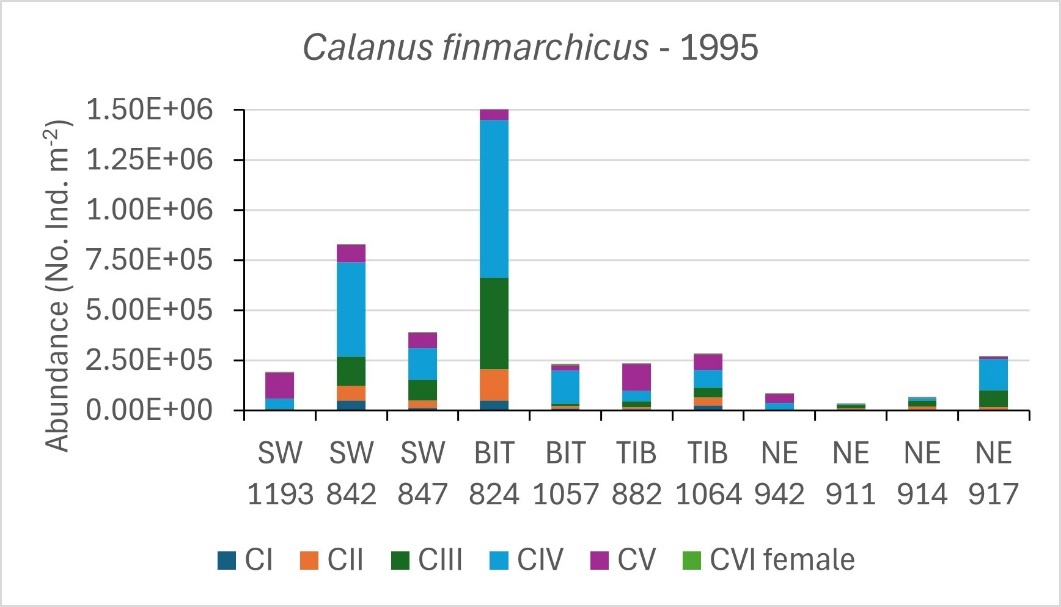


Fig. S-10. Copepodite stage composition of *Calanus finmarchicus* at stations in three Atlantic polygons (SW, BIT, TIB) and the NE polygon in 1995.


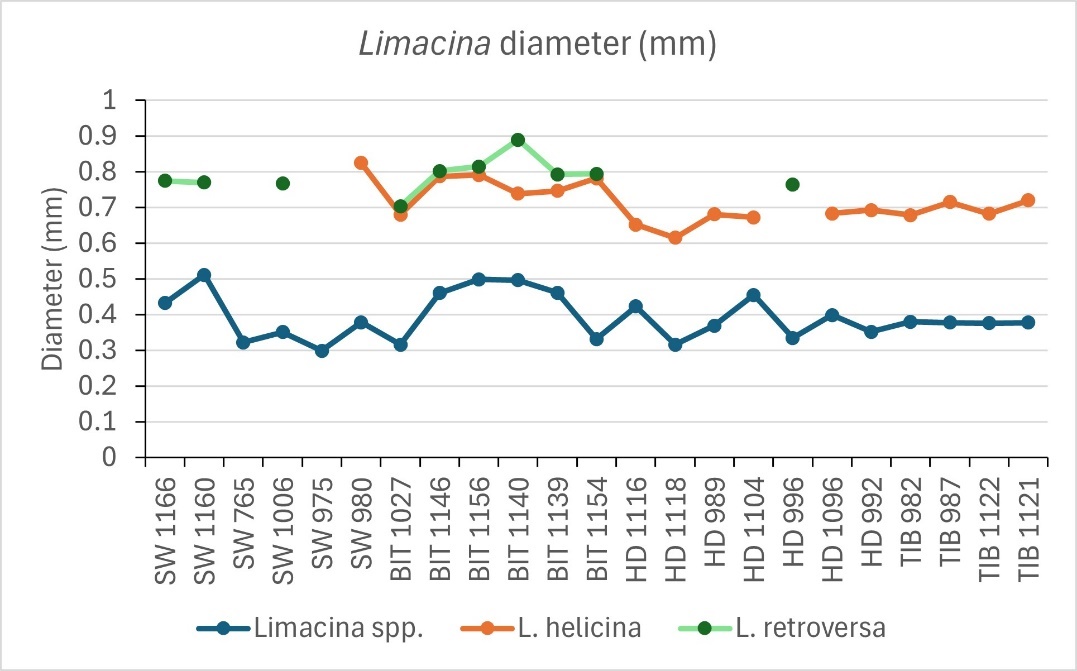


Fig. S-11. Mean size (diameter) of small, unidentified juvenile *Limacina* spp., and larger (>~0.6 mm) juveniles identified as *L. helicina* and *L. retroversa* at the stations sampled in 1994.


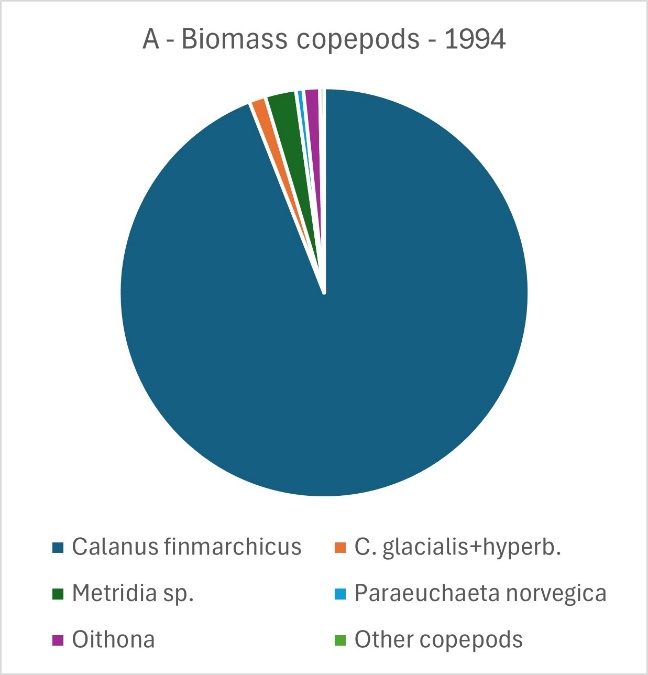

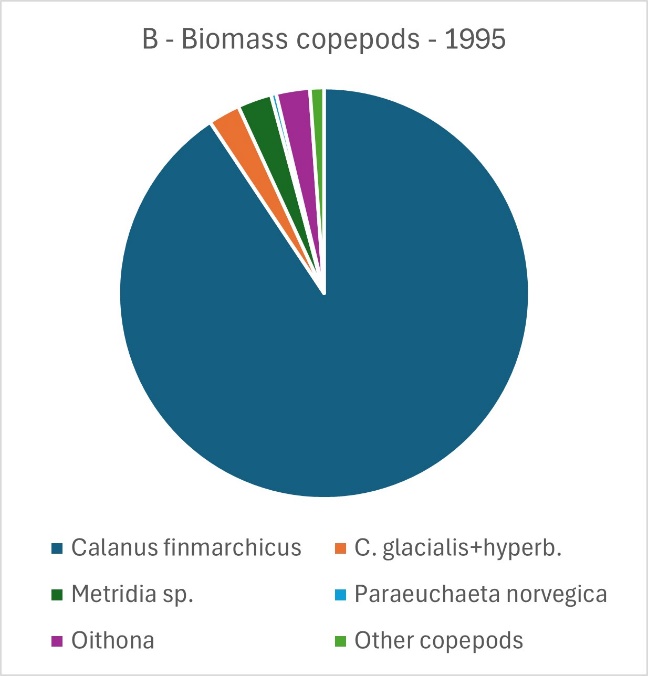


Fig. S-12. Biomass composition of copepods in 1994 and 1995 based on estimated biomass calculated from abundance and weights for individuals of species and copepodite stages. Average values for the analyzed samples from 23 stations in 1994 and 11 stations in 1995. The estimated total biomass for copepods were 22.2 and 22.7 g dw m^-2^ in 1994 and 1995, respectively (Table S-6).


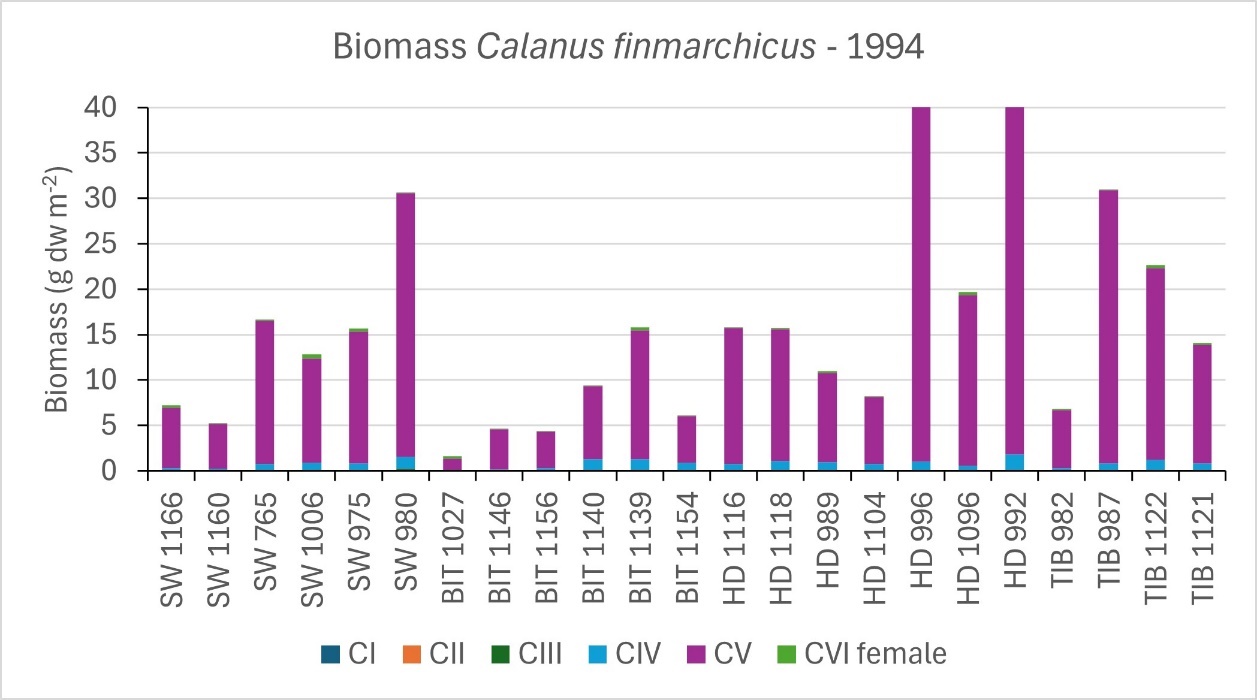


Fig. S-13. Estimated biomass calculated from abundance and individual weights of copepodite stages C1 to C6 of *Calanus finmarchicus* for the analyzed samples from sampling stations in 1994. The stations are identified by the four Atlantic polygons (SW, BIT, HD and TIB). The scale is cut at 40 g dw m^-2^ and does not show the two highest values of 94 and 102 g dw m^-2^ for stations 996 and 992 in the HD polygon.


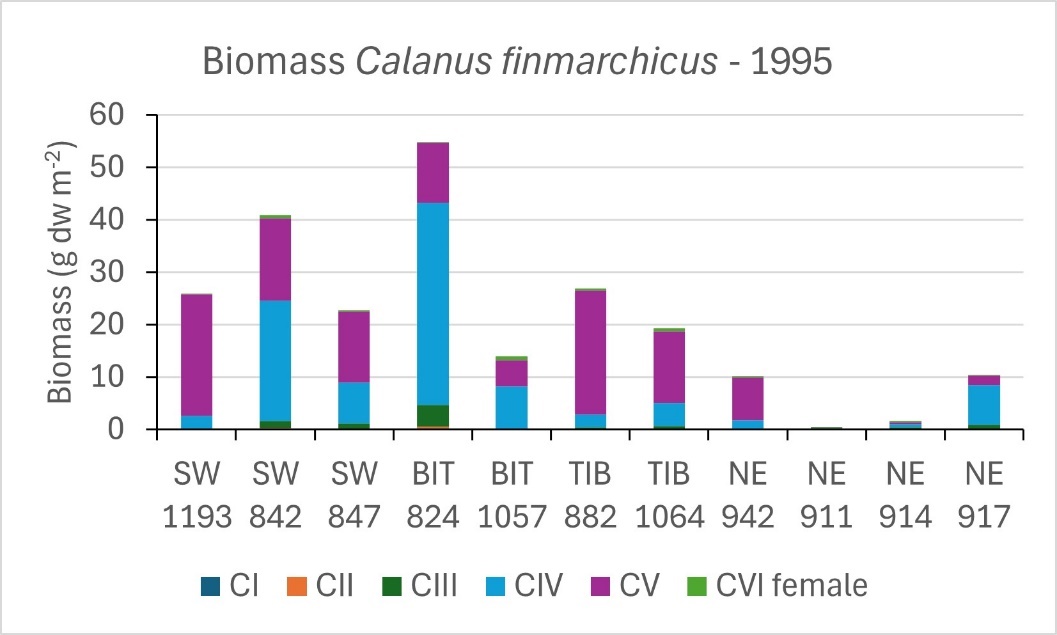


Fig. S-14. Estimated biomass calculated from abundance and individual weights of copepodite stages C1 to C6 of *Calanus finmarchicus* for the analyzed sampling stations in 1995. The stations are identified by three Atlantic polygons (SW, BIT, and TIB) and the NE polygon (see map in Fig. S-1).


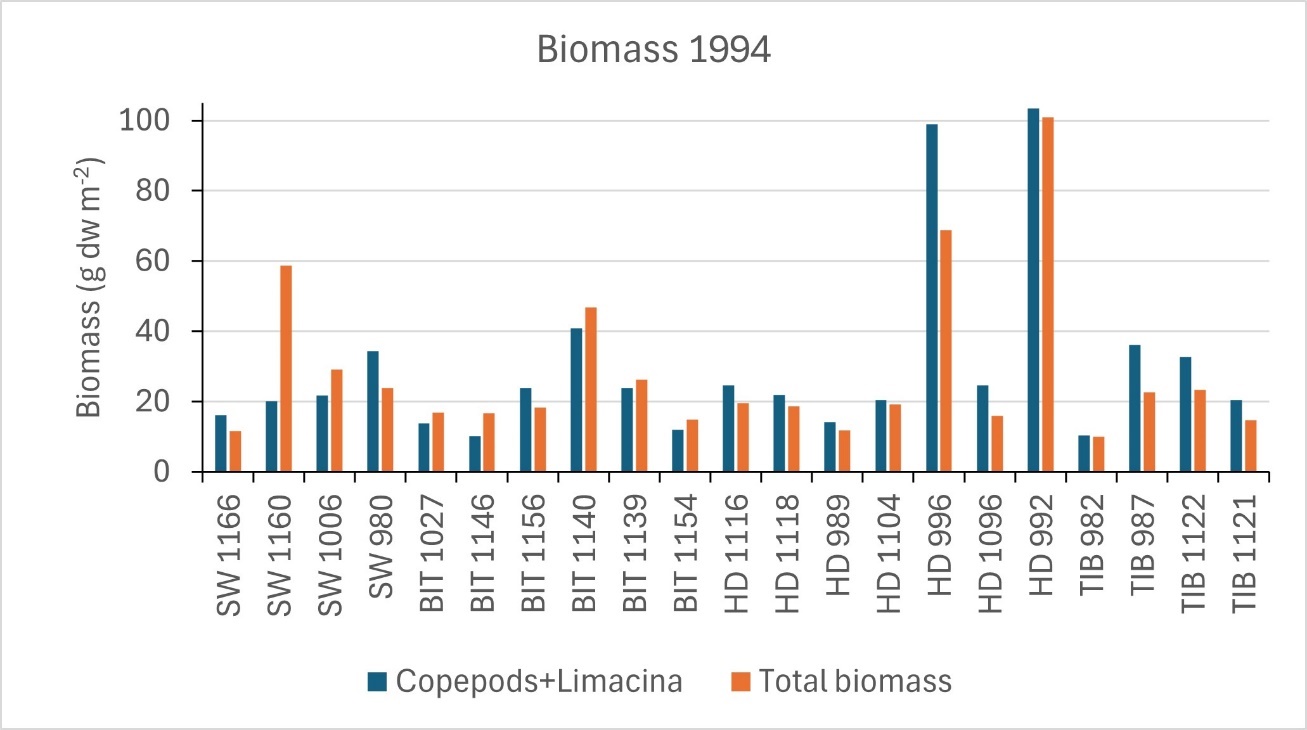


Fig. S-15. Sum of estimated biomass of copepods and *Limacina* pteropods (calculated from numbers) and directly measured total zooplankton biomass across 23 sampling stations in the four Atlantic polygons (SW, BIT, HD and TIB) in 1994.


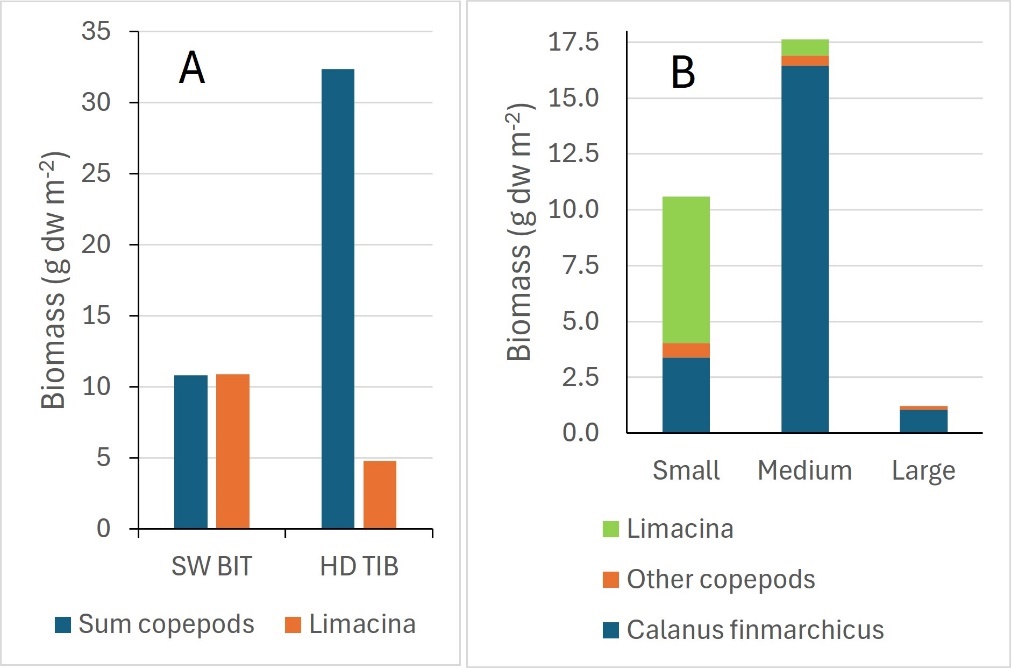


Fig. S-16. Estimated biomass of copepods and *Limacina* pteropods (calculated from numbers) for sampling stations in 1994. A. Mean biomass values for two sets of Atlantic polygons – SW and BIT, and HD and TIB (see Fig. S-1 for location of polygons). B. Mean estimated biomass values for *Calanus finmarchicus*, other copepods, and *Limacina* spp. allocated to three size fractions (small <1 mm, medium 1-2 mm, large >1 mm).


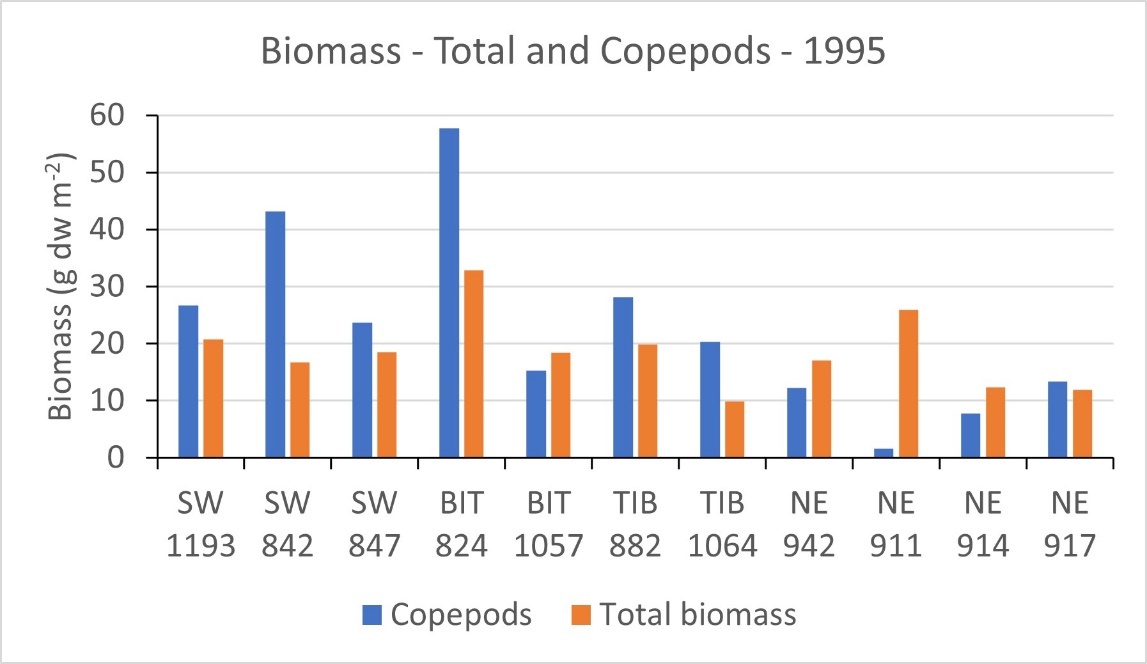


Fig. S-17. Sum of estimated biomass of copepods (calculated from numbers) and directly measured total zooplankton biomass across 11 sampling stations in three of the Atlantic polygons (SW, BIT, and TIB) and in the NE polygon in 1995. See Fig. S-1 for location of polygons.
